# Interpretable design of Ir-free trimetallic electrocatalysts for ammonia oxidation with graph neural networks

**DOI:** 10.1038/s41467-023-36322-5

**Published:** 2023-02-11

**Authors:** Hemanth Somarajan Pillai, Yi Li, Shih-Han Wang, Noushin Omidvar, Qingmin Mu, Luke E. K. Achenie, Frank Abild-Pedersen, Juan Yang, Gang Wu, Hongliang Xin

**Affiliations:** 1 Department of Chemical Engineering, Virginia Polytechnic Institute and State University, Blacksburg, VA USA; 2 School of Materials Science and Engineering, Jiangsu University, Zhenjiang, Jiangsu China; 3 SUNCAT Center for Interface Science and Catalysis, SLAC National Accelerator Laboratory, Menlo Park, CA USA; 4 Department of Chemical and Biological Engineering, University at Buffalo, The State University of New York, Buffalo, NY USA

**Keywords:** Materials for energy and catalysis, Catalytic mechanisms, Electrocatalysis, Theoretical chemistry

## Abstract

The electrochemical ammonia oxidation to dinitrogen as a means for energy and environmental applications is a key technology toward the realization of a sustainable nitrogen cycle. The state-of-the-art metal catalysts including Pt and its bimetallics with Ir show promising activity, albeit suffering from high overpotentials for appreciable current densities and the soaring price of precious metals. Herein, the immense design space of ternary Pt alloy nanostructures is explored by graph neural networks trained on ab initio data for concurrently predicting site reactivity, surface stability, and catalyst synthesizability descriptors. Among a few Ir-free candidates that emerge from the active learning workflow, Pt_3_Ru-M (M: Fe, Co, or Ni) alloys were successfully synthesized and experimentally verified to be more active toward ammonia oxidation than Pt, Pt_3_Ir, and Pt_3_Ru. More importantly, feature attribution analyses using the machine-learned representation of site motifs provide fundamental insights into chemical bonding at metal surfaces and shed light on design strategies for high-performance catalytic systems beyond the *d*-band center metric of binding sites.

## Abstract (web summary)

Electrochemical conversion of ammonia to nitrogen has important energy and environmental applications but is hindered by lack of efficient electrocatalysts. Here the authors use quantum chemistry and machine learning to gain insights into the reaction mechanism and accelerate the design of highly active Ir-free trimetallic catalysts.

## Introduction

Ammonia (NH_3_), an essential molecule of the planetary nitrogen cycle, has been widely used in modern industries ranging from medicine, food, and agriculture to the manufacturing of household chemicals. Particularly promising is its application for energy generation and storage technologies because of its easy liquefaction and high volumetric energy density, appealing characteristics for coping with the variability of renewable energy sources, e.g., wind, solar, and hydro^1^. In this aspect, design of materials for efficiently catalyzing the ammonia oxidation reaction (AOR) at the anode of direct ammonia fuel cells (DAFCs) holds the key to enable the transition away from fossil feedstocks to a sustainable energy economy. Platinum (Pt) is known as the best elemental metal catalyst for NH_3_ oxidation to benign dinitrogen (N_2_) in alkaline media, albeit suffering from large overpotentials (~0.5 V)^2,3^. Iridium (Ir) exhibits a lower onset potential than Pt attributed to a stronger interaction with nitrogen species, which however leads to site poisoning at high operating potentials. Experimental studies on Pt single-crystal electrodes showed that the reaction is structure sensitive with {100}-type site motifs being dominantly active^4,5^. In situ spectroscopic characterization and quantum-chemical simulations^3^ suggest that NH_3_ electrooxidation on Pt at low operating potentials likely proceeds through the Gerischer-Mauerer mechanism^6^, in which partial dehydrogenation of NH_3_ to *NH_*x*_ (*x* = 1 or 2) is followed by their dimerization to *N_2_H_*y*_ and further dehydrogenation of *N_2_H_*y*_ to N_2_, yet complete dehydrogenation of NH_3_ to *N gives rise to surface deactivation, presumably by strongly adsorbed *N or its oxidatively derived species, e.g., *NO^2,7^. Alloying Pt with Ir effectively lowers the onset potential of sluggish dehydrogenation steps while allowing the dimerization of nitrogen species at reasonable temperatures^8–10^. To enhance the process efficiency and economic viability for large-scale applications, there have been several efforts in exploring multimetallic Pt alloys with earth-abundant elements^11–14^. However, a lack of detailed kinetic understanding of electrochemical reactions and the vast compositional space for materials screening make it difficult to find improved AOR electrocatalysts through trial and error.

From a computational perspective, screening of catalytic materials with desired properties, e.g., moderate adsorption energies of descriptor species underpinned by the Sabatier principle^15^, is a highly complex, multidimensional optimization process. By modulating rate-determining bond breaking or formation steps, theory-guided design of active sites has been pursued for decades in catalysis^16–18^. Due to the intrinsic complexity of chemical bonding at solid surfaces^19^, uncovering underlying factors that govern the effectiveness of heterogeneous catalysts still remains a formidable task. With recent advances in data infrastructure and software frameworks, machine learning (ML) has emerged as a promising tool for accelerating catalytic materials discovery in many energy and environmental technologies, e.g., H_2_O oxidation^20,21^, CO_2_ reduction^22^, ${{{\rm{NO}}}}_{3}^{-}$ reduction^23^, H_2_ evolution^24^, and O_2_ reduction^25^. By learning from ab initio data, ML surrogate models that map the feature representation of site motifs onto their catalytic properties^26,27^ can iteratively narrow down candidate catalysts prior to laborious experimental synthesis, characterization, and performance testing. However, a looming obstacle of deploying purely data-driven ML solutions in catalysis, particularly with the resurgent deep learning algorithms, is their poor interpretability, which prevents us from drawing physical insights that can be translated into design of hierarchical systems not included in the hypothesis space.

By leveraging emerging developments of computational and data sciences, the design space of ternary Pt alloy nanostructures was thoroughly explored for promising AOR electrocatalysts. With descriptor-based microkinetic modeling, the theoretical activity trends of preferable {100}-type site motifs were mapped onto the bridge and hollow N adsorption energies as two reactivity descriptors that are linearly correlated with the free formation energies of AOR intermediates and transition states. Nonetheless, the immense design space spanned by the crystal structure and metal identity of bimetallics together with the atomistic configuration of a third-metal component makes it too time-consuming to search for optimal electrocatalysts using high-throughput density functional theory (DFT) calculations. Herein, deep learning with graph feature representations, e.g., graph neural networks^28^, is used in an active learning workflow for concurrent predictions of site reactivity, surface stability, and catalyst synthesizability descriptors, drastically reducing the number of required DFT calculations. The screening is enabled by theory-infused neural networks (TinNet) to not only identify promising electrocatalysts but also uncover underlying factors governing reactivity properties of site motifs^29^. Among a few Ir-free candidates, Pt_3_Ru-M (M: Fe, Co, or Ni) alloy nanocubes were successfully synthesized and experimentally verified to be more active toward AOR than Pt, Pt_3_Ir, and Pt_3_Ru. More importantly, feature attribution analyses provides fundamental insights into physical factors, e.g., adsorbate resonance energies of frontier orbitals, governing reactivity properties of *d*-metal alloy surfaces and shed light on design strategies for high-performance catalytic systems beyond the *d*-band center metric of binding sites.

## Results

### Microkinetic modeling of AOR on metal surfaces

**Fig. 1:**
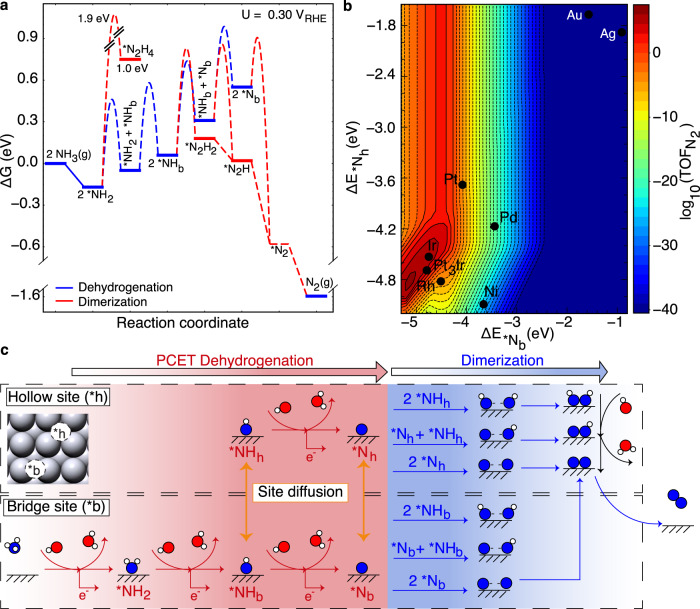
Reaction pathways of NH_3_ electrooxidation and the activity volcano plot. **a** Free energy profiles of electrochemical NH_3_ oxidation to N_2_ on Pt(100) at 0.3 V vs. RHE from grand-canonical DFT calculations. **b** The activity volcano plot of the NH_3_ electrooxidation as a function of the bridge and hollow N adsorption energies on {100}-type site motifs (reaction conditions: 0.3 V vs. RHE, *T* = 298 K, pH = 14, and *c*_NH3_ = 0.1 M). A few transition and noble metals along with Pt_3_Ir are marked on the map. **c** Adsorption configurations of key intermediates within the complex reaction network used in microkinetic modeling (Pt: light gray, N: blue, O: red, H: white).

Figure 1a shows the free energy profiles of NH_3_ electrooxidation pathways to N_2_ on Pt(100) from grand-canonical DFT calculations at 0.3 V vs. RHE (see Methods for computational details and Supplementary Tables 1 and 2 for energetics). We chose the potential for screening at 0.3 V vs. RHE, which would be the most optimistic target similar to other reactions, e.g., oxygen evolution, oxygen reduction. Another consideration is the difficulty of modeling electrokinetics at high potentials, under which the deactivation of surface sites occurs as evidenced by chronoamperometry (CA). The mechanistic pathway that leads to site poisoning likely by *NO remains elusive. The potential-dependent activation barriers of proton-coupled electron transfer reactions were obtained using the approach by Nie and Janik^30^ with the symmetry factor set to 0.5. *OH is considered as a coadsorbed species because of its favorable formation on Pt(100) at ~0.3 V vs. RHE^31^. As can be seen in Fig. 1a, the dehydrogenation steps of *NH_2_ to *NH and then to *N are both endergonic with a free energy barrier of ~0.6 eV. For the dimerization of nitrogen species, only the *NH and *N pathways were included because the bridge *NH_2_ is kinetically prohibited to dimerize at the coverage of 1/2 ML or less (~1.9 eV barrier). *NH_2_ dimerization on Pt(100) becomes possible at a coverage of 0.75 ML when *NH_2_ prefers an atop adsorption configuration^32^ (Supplementary Fig. 1), rendering the high *NH_2_ coverage inaccessible. In light of facile dimerization of *N species on Pt(100) as shown in Fig. 1a, *N might not be the poisoning species as previously assumed. Instead, the coupling of *N with *OH can lead to the formation of thermodynamically stable *NO that deteriorates the electrode activity^2,7,33^. Figure 1b shows the theoretical AOR activity map at 0.3 V vs. RHE (close to the measured onset potential of Pt) from microkinetic modeling using the reaction pathways in Fig. 1a and the linear free energy scaling relationships on {100} *d*-metal surfaces (Supplementary Fig. 2), with the bridge and hollow N adsorption energies, $\varDelta {E}_{*{{{{{{\rm{Nb}}}}}}}}$ and $\varDelta {E}_{*{{{{{{\rm{Nh}}}}}}}}$, as two reactivity descriptors. To avoid unrealistic surface coverages of key intermediates, a coverage-dependent formation energy of *NH_2_ is built into microkinetics (Supplementary Fig. 3a). Inclusion of coverage-dependent energetics for *NH has no influence on the overall activity trends, thus ignored for simplicity (Supplementary Fig. 3b). A few late transition and noble metals along with Pt_3_Ir (the most stable termination with a mixed surface layer of Pt/Ir) are marked on the map. It can be seen that Ir and Pt_3_Ir are more active than Pt at 0.3 V vs. RHE, which agrees very well with the experimental observation that Ir reduces the onset potential of AOR by adsorbing *NH_2_ strongly to form *NH and *N intermediates that can dimerize with activation barriers surmountable at reasonable temperatures^14^. However, at high operating potentials (>0.5 V vs. RHE), Ir exhibits a lower current density than Pt likely because of the formation of poisoning species (e.g., *NO), the underlying mechanism of which is not well understood. The activity volcano map suggests that to design electrocatalysts with improved catalytic performance than Pt_3_Ir one needs to find site motifs that bind *N stronger while weakly enough for a balance of dehydrogenation and dimerization kinetics^34^.

### Machine learning prediction of Ir-free ternary Pt alloys for AOR

**Fig. 2:**
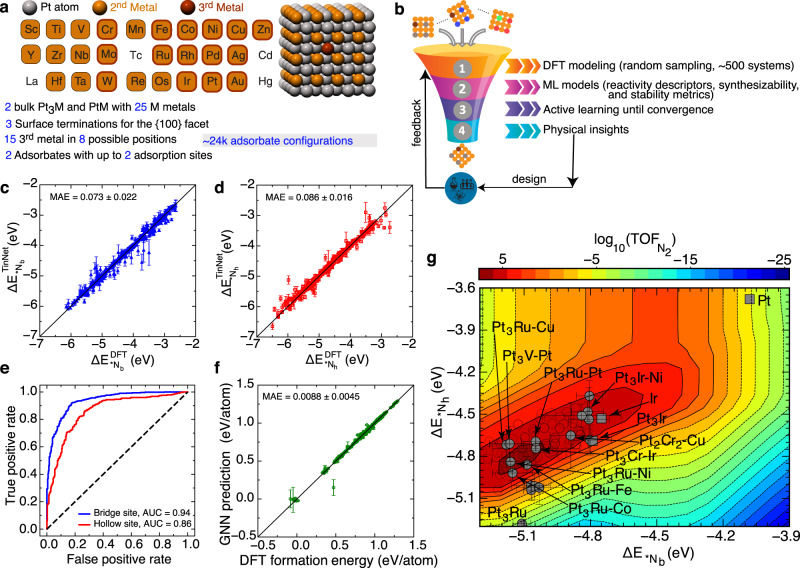
Screening of trimetallic Pt alloy electrocatalysts for AOR with graph neural networks. **a** The immense design space of trimetallic Pt nanocatalysts terminated with {100} facets. **b** An active learning workflow for accelerating catalytic materials discovery. **c**–**f** The 10-fold final models of graph neural networks for predicting site reactivity, surface stability, and catalyst synthesizability descriptors. **g** The AOR activity map at 0.3 V vs. RHE with solid markers showing promising ternary Pt alloy electrocatalysts predicted from the workflow. Error bars indicate the standard deviation in the adsorption-energy predictions from 10-fold cross-validation. Square markers represent Pt, Ir, and Pt_3_Ir electrocatalysts. Open markers are systems with theoretical activity higher than Pt_3_Ir albeit not passing the stability and synthesizability filters.

To go beyond Pt and Pt_3_Ir electrocatalysts, it is a natural step to explore ternary Pt alloys with an improved activity and possibly reduced use of precious metals. Figure 2a depicts the design space of ternary Pt alloys with {100}-type site motifs. This space was constructed by firstly curating fcc crystal structures of Pt_3_M and PtM intermetallics from the Materials Project^35^, where M can be any of the 25 metals. Subsequently, {100}-oriented surface terminations of those bimetallics were cleaved, and trimetallic surfaces were automatically generated by replacing an atom of the top-two layers with a third-metal atom (one of the 15 metal choices in Fig. 2a). The effect of substituting a metal in the third and fourth layer had a negligible effect on the binding energy (Supplementary Table 3). Atomistic configurations were then enumerated for *N_b_ and *N_h_ with 1/4 ML *OH coadsorbed at a neighboring bridge site. This leads to ~24k unique structural models, which makes it too time-consuming to compute directly from first principles. In the data-driven design workflow as shown in Fig. 2b, ~500 systems were randomly sampled from the hypothesized materials space and DFT calculations were performed to obtain N adsorption energies. A system is deemed as stable if the *N and *OH coadsorbed surface does not reconstruct during geometry optimization (if position changes <0.5 Å for all metal atoms). For the synthesizability of an atomistic configuration, the formation energy of 1/4 ML *OH is computed by referencing to the metal bulk phases, H_2_O(*l*), and H_2_(*g*). Those data were used to train regression and classification ML models for quantifying site reactivity, surface stability, and catalyst synthesizability descriptors (see Methods). Particularly, the theory-infused neural network (TinNet) was adopted for predicting surface reactivity^29^, i.e., the bridge and hollow N adsorption energies. TinNet is an integration of graph neural networks (GNNs) with a theory module in architecture design for domain-specific interpretations (see Supplementary Fig. 4 for the architecture of graph neural networks). For chemical bonding at metal surfaces, the theory module is implemented to employ the Newns–Anderson-type Hamiltonians^36^ for describing the energetic and electronic evolution of an adsorption process with physical factors represented by neural state variables from the regression module. It is important to emphasize that those variables, representing adsorbate resonance energy, interatomic coupling coefficient, and other physical factors embedded in TinNet models, are latent variables that cannot be easily tabulated but can be learned from data using convolutional operations. While highly complex, the non-linearity of GNNs is key in accurately describing the binding energies, in which simple linear models albeit interpretable struggle tremendously (Supplementary Fig. 5). For surface stability and catalyst synthesizability descriptors, GNNs were used directly for classifying whether a surface is stable against reconstruction by *N adsorbates and predicting the formation energy of 1/4 ML *OH, respectively. To balance the exploration and exploitation in screening, a new set of systems will be selected based on the predicted mean and its uncertainty from the nested 10-fold cross-validation. Specifically, material systems with a high likelihood (> 80%) of surface stability can pass through the filter. Subsequently, atomistic configurations for each composition are sorted by the formation energy and those most stable systems within one standard deviation of model errors are considered synthesizable. Lastly, all systems will be ranked by the predicted AOR activity, and the top 75 candidates are chosen in a new iteration for DFT calculations if not already done. Those data will be updated into the database to retrain all the ML models. The process is repeated till convergence, defined as the point where no additional system with activity higher than Pt_3_Ir can be identified. Figure 2c–f show the 10-fold final models with high prediction accuracies for site reactivity, surface stability, and catalyst synthesizability descriptors after 2 iterations of active sampling of the design space (Supplementary Figs. 6–10, Table 4, 5). Figure 2g highlights several of the systems that emerge from the screening process with the theoretical activity higher than Pt_3_Ir while being Ir-free, for example Pt_3_Ru–M (M: Fe, Co, or Ni), making them highly desirable for experimental synthesis.

### Controlled synthesis and characterization of predicted electrocatalysts

**Fig. 3:**
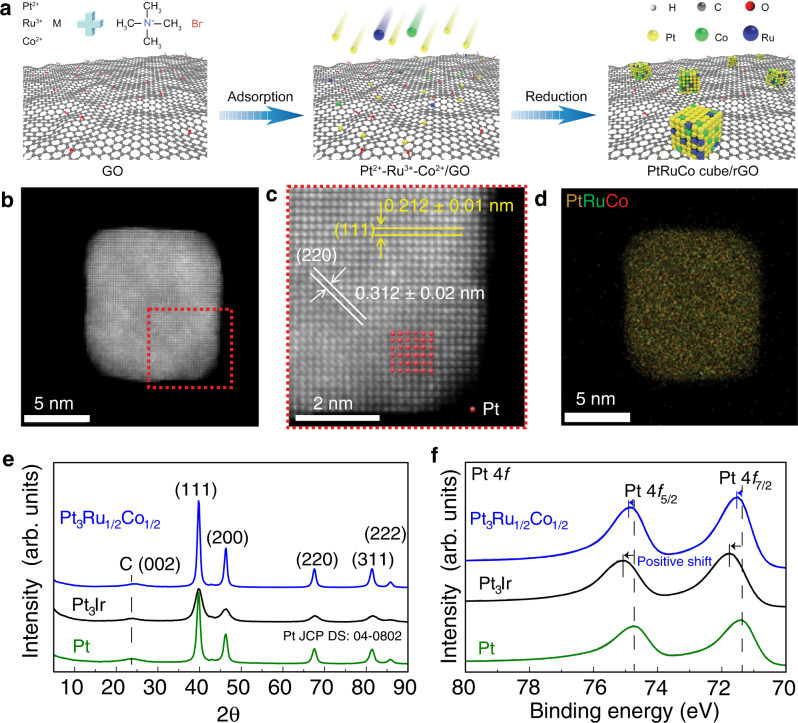
Structural and compositional analyses of Pt_3_Ru_1/2_Co_1/2_ alloy electrocatalysts. **a** Schematic of the Pt_3_Ru_1/2_Co_1/2_ ternary alloy synthesis, **b**–**d** HAADF-STEM images and the corresponding EDS elemental mapping of Pt, Ru and Co. **e** XRD patterns and (**f**) XPS spectra of the as-prepared electrocatalysts. Pt and Pt_3_Ir catalysts were used as references.

Among those predicted to be active, we have successfully synthesized Pt_3_Ru_1/2_Co_1/2_ ternary alloy nanocubes as shown in Fig. 3a (see Methods). The overall morphology and particle distribution of the ternary Pt_3_Ru_1/2_Co_1/2_ alloy nanoparticles on reduced graphene oxide (rGO) surfaces were characterized by scanning electron microscopy (SEM, Supplementary Fig. 11) and transmission electron microscopy (TEM, Supplementary Fig. 12). The total metal (i.e., Pt, Ru, and Co) content measured by inductively coupled plasma mass spectrometry (ICP-MS) is about 18.6 wt%, similar to the precursor used, indicating an almost complete reduction and growth of the ternary alloy nanoparticles on rGO surfaces.

To gain insights into the local structure of the alloy nanoparticles, we conducted high-angle annular dark-field-scanning transmission electron microscopy (HAADF-STEM) and EDS measurements. The STEM images showed the cubic nanoparticle with a particle diameter of 10.0 ± 0.2 nm (Fig. 3b), and the enlarged image (in red circle, Fig. 3c) displays the atomic structure of the surface region of Pt_3_Ru_1/2_Co_1/2_ with the lattice fringes of 0.212 ± 0.01 nm at the lateral direction and 0.312 ± 0.02 nm at the angular direction, corresponding to (111) and (220) planes of Pt_3_Ru_1/2_Co_1/2_ alloys, respectively. These lattice fringes are slightly smaller than that of the standard pure Pt (JCPDS: 04-0802), with the corresponding lattice fringes of 0.221 nm and 0.360 nm, respectively. Figure 3d shows the overlay of elemental mapping images, where the atom columns of Pt, Ru and Co are also clearly resolved in Supplementary Figs. 14, 15. Energy-dispersive X-ray spectroscopy line scanning further demonstrates the uniform distribution of Pt, Ru, and Co atoms in nanostructures (EDS, Supplementary Fig. 16).

The X-ray diffraction (XRD) patterns in Fig. 3e show broad diffraction peaks at ca. 23.8°, assigning to the (002) facet of carbon from the reduced graphene oxide^37^. The diffraction peaks of the pure Pt catalyst can be identified at ca. 39.8, 46.2, 67.5, 81.3 and 85.7°, corresponding to the (111), (200), (220), (311) and (222) facets of face centered cubic crystalline Pt (JCPDS: 04-0802). Compared to the pure Pt catalyst, these diffraction peaks in Pt_3_Ir and Pt_3_Ru_1/2_Co_1/2_ catalysts show slightly positive shift because Pt atoms were partially replaced by smaller Ir or Ru/Co atoms, leading to a lattice compression of the nanoparticles^13,14^. These results further confirm the formation of Pt_3_Ir and Pt_3_Ru_1/2_Co_1/2_ alloy nanoparticles. X-ray photoelectron spectroscopy (XPS) was conducted to identify the near surface elemental compound and chemical state. High-resolution XPS of Pt 4 f in rGO-supported Pt_3_Ir and Pt_3_Ru_1/2_Co_1/2_ (Fig. 3f) shows that the binding energy of Pt 4 f shifts to a higher value relative to rGO-supported Pt, which can be attributed to the strong electronic interaction within the Pt_3_Ir and Pt_3_Ru_1/2_Co_1/2_^38^.

**Fig. 4:**
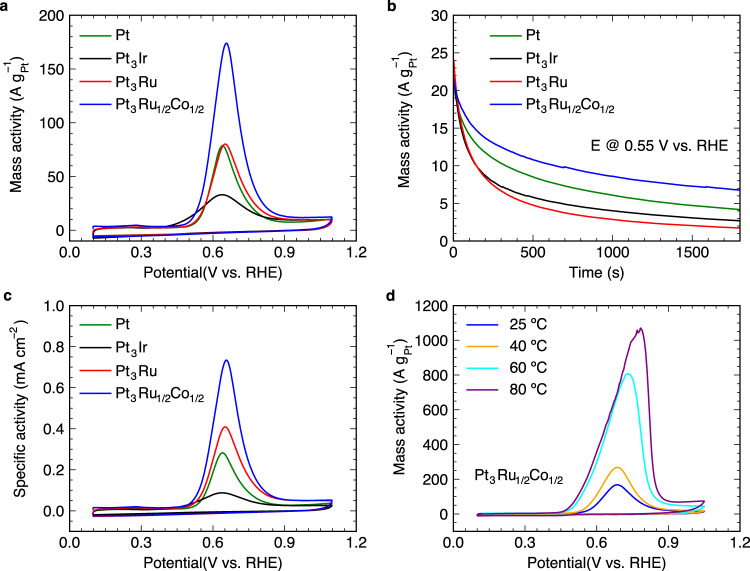
NH_3_ electrooxidation performance. **a** Electrocatalytic performance testing of the Pt, Pt_3_Ir, Pt_3_Ru and Pt_3_Ru_1/2_Co_1/2_ nanocubes via cyclic voltammetry (CV) with a rotating speed of 900 rpm in Ar-saturated 1.0 M KOH + 0.1 M NH_3_ under ambient conditions. **b** The NH_3_ electrooxidation short-term stability of the Pt, Pt_3_Ir, Pt_3_Ru and Pt_3_Ru_1/2_Co_1/2_ nanocubes at 0.55 V vs. RHE using the same testing conditions. **c** Specific activity comparisons for the Pt, Pt_3_Ir, Pt_3_Ru and Pt_3_Ru_1/2_Co_1/2_ nanocubes. **d** Cyclic voltammetry curves for the Pt_3_Ru_1/2_Co_1/2_ in Ar-saturated 1.0 M KOH + 0.1 M NH_3_ electrolytes operating at temperatures of 25, 40, 60 and 80 °C with a scanning rate of 5 mV s^–1^ and rotating speed of 900 rpm.

Catalytic performance of the Pt, Pt_3_Ir, Pt_3_Ru and Pt_3_Ru_1/2_Co_1/2_ nanocubes were tested via cyclic voltammetry (CV) in an Ar-saturated 1.0 M KOH + 0.1 M NH_3_ aqueous electrolyte (Pt_3_Ru was included as a reference system). Figure 4a shows that Pt_3_Ir has the lowest onset potential of 0.35 V vs. RHE, while Pt_3_Ru and Pt_3_Ru_1/2_Co_1/2_ have onset potentials of 0.46 and 0.42 V, all of which are lower than pure Pt of 0.48 V. Nevertheless, Pt_3_Ru_1/2_Co_1/2_ has the highest peak mass current activity of 174.0 A g^–1^_Pt_, higher than those of pure Pt (78.6 A g^–1^_Pt_), Pt_3_Ir (33.1 A g^–1^_Pt_) and Pt_3_Ru (80.2 A g^–1^_Pt_) catalysts. Further CO stripping measurements in CO-saturated 0.1 M HClO_4_ aqueous solution were conducted to estimate the electrochemical activate area (ECSA) of those Pt-based catalysts (see Supplementary Fig. 17). The ECSA values for Pt, Pt_3_Ir, Pt_3_Ru and Pt_3_Ru_1/2_Co_1/2_ were estimated to be 27.8, 37.7, 19.6 and 23.7 m^2^ g^–1^, respectively, which were summarized in Supplementary Fig. 18. Accordingly, the AOR specific activities for those Pt-based catalysts were plotted in Fig. 4c, showing a similar activity trend as the mass activity. The temperature dependent AOR activity of the rGO-supported Pt_3_Ru_1/2_Co_1/2_ catalyst was further measured, as shown in Fig. 4d. With increasing temperature from 25 to 80 °C, the AOR current significantly increased, resulting in lowered onset potentials and six-fold enhancement of the peak current density. In addition, the synthesized rGO-supported Pt_3_Ru_1/2_Ni_1/2_ and Pt_3_Ru_1/2_Fe_1/2_ nanocubes (Supplementary Fig. 19, 20) also show more active AOR performance than Pt, Pt_3_Ir, and Pt_3_Ru (Supplementary Fig. 21). For example, the highest peak current densities for Pt_3_Ru_1/2_Ni_1/2_ and Pt_3_Ru_1/2_Fe_1/2_ are 123.0 and 181.4 A g^–1^_Pt_, respectively. These activity trends correspond well with the prediction trend from the screening shown in Fig. 2g, further validating the design approach. It is important to note that the adsorption energies of both *N bridge and hollow species on Pt_3_Ru_1/2_Ni_1/2_, Pt_3_Ru_1/2_Fe_1/2_ and Pt_3_Ru_1/2_Co_1/2_ are within 0.1 eV, which is similar to the uncertainty of the microkinetic models introduced by linear adsorption-energy scaling relations. The short-term AOR stability of the Pt, Pt_3_Ir, Pt_3_Ru and Pt_3_Ru_1/2_Co_1/2_ nanocubes was studied via chronoamperometry (CA) at 0.55 V vs. RHE in an Ar-saturated 1.0 M KOH + 0.1 M NH_3_ aqueous electrolyte. As shown in Fig. 4b, a rapid decline in current density is noticed during the CA running. The initial rapid decline is derived from the removal of non-faradaic double-layer currents, while the subsequent decay is due to the deactivation of surface sites^37,39,40^. This is likely attributed to the formation of poisoning species such as *NO that blocks the active sites^40,41^. Compared to pure Pt, Ir alloying results in an enhanced AOR activity (Fig. 4a), while Ru does not improve the activity and shows a faster activity loss during the CA running (Fig. 4b). Encouragingly, the introduction of Co tends to stabilize surface sites in Pt_3_Ru_1/2_Co_1/2_, and showed ~4 times higher remained current than Pt_3_Ru, further indicating the critical role of both Ru and Co atoms in the ternary alloy system. Specifically, the Pt_3_Ru_1/2_Co_1/2_ retained the highest current density (6.7 A g^–1^_Pt_) after 1,800 s of CA running when compared with Pt_3_Ir (5.6 A g^–1^_Pt_), Pt_3_Ru (1.7 A g^–1^_Pt_) and Pt (4.2 A g^–1^_Pt_). Furthermore, their electrochemical surface area changes before and after 1800 s of CA testing was studied and shown in Supplementary Fig. 22, which further confirmed the superiority of the ternary Pt_3_Ru_1/2_Co_1/2_ toward AOR electrocatalysis. Most importantly, those catalysts are Ir-free, which is an important step toward potentially large-scale commercial applications of direct ammonia fuel cells.

### Advancing theory of chemisorption with interpretable deep learning

**Fig. 5:**
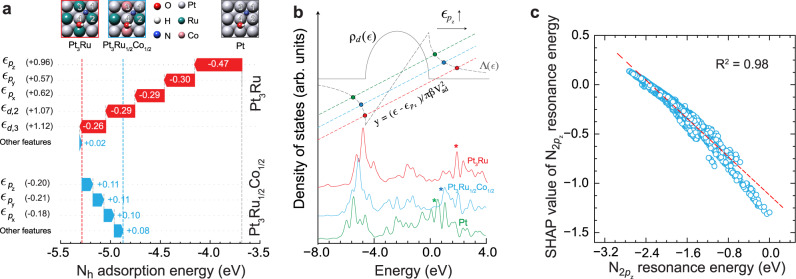
Physicochemical insights from interpretable deep learning. **a** Local interpretation of N_h_ adsorption on Pt_3_Ru(100) relative to Pt(100) and Pt_3_Ru_1/2_Co_1/2_(100) to Pt_3_Ru(100). **b** A graphical illustration of the Newns-Anderson model for an adsorbate resonance state interacting with metal *d*-states. DFT-calculated DOS projected onto the 2*p*_z_ orbital of *N_h_ on Pt_3_Ru_1/2_Co_1/2_, Pt_3_Ru, and Pt are shown with adsorbate-metal antibonding states highlighted for comparison. **c** Global SHAP analysis of TinNet models of N_b_ adsorption showing a strong correlation of the machine-learned adsorbate resonance energy with the adsorption-energy contribution, i.e., the SHAP value.

To gain physical insights into the superior AOR activity of Pt_3_Ru–M (M: Fe, Co, or Ni) electrocatalysts, we have inspected the causal relationships between the machine-learned features of a site motif and reactivity descriptors using post hoc interpretation techniques. This type of fundamental interrogation of deep learning models is enabled by the interpretable nature of the TinNet framework that embeds a physically transparent, data-derived mapping from neural state variables to output targets. We used the Shapley Additive exPlanations (SHAP)^42–44^ that are built upon cooperative game theory for a local interpretation of reactivity origin of metal sites, by computing the SHAP value of each machine-learned feature, i.e., feature contribution to a prediction relative to a baseline or reference (see Methods). As shown in Fig. 5a, a local interpretation of Pt_3_Ru relative to Pt and Pt_3_Ru_1/2_Co_1/2_ to Pt_3_Ru, taking *N_h_ as an example, suggests that the adsorbate resonance energies of the frontier N_2*p*_ orbitals are the most important factors in governing site reactivity instead of the commonly employed *d*-band center metric. Even though the *d*-band center of the Ru atom 2 and 3 in the Pt-Ru ensemble relative to pure Pt shifts up by +1.07 and +1.12 eV, respectively, it only leads to a –0.55 eV energy stabilization. In contrast, the adsorbate resonance energy of the *N 2*p*_*z*_ (2*p*_*x*_ and 2*p*_*y*_) orbital shifts up by +0.96 (+0.56 and +0.62) eV at Pt_3_Ru relative to that at Pt, which contributes –0.47 (–0.30 and –0.29) eV stabilization to N_h_ adsorption. Interestingly, the 3^rd^ metal Co at the atom site 3 (most stable position) weakens N_h_ adsorption by slightly shifting down the resonance energies of the N_2*p*_ orbitals, giving near-optimal reactivity descriptors. Local interpretable model-agnostic explanations (LIME) analysis was also performed, showing consistent interpretation where the variations in binding strengths can be largely explained due to the changes in the adsorbate resonance energies of frontier orbitals (Supplementary Fig. 23). Nevertheless, the factors that govern the surface reactivity, i.e., adsorption energies, are system dependent. In the case of Pt, Pt_3_Ru, and Pt_3_Ru_1/2_Co_1/2_, the variation of N_h_ adsorption energies can be explained by the resonance energies of adsorbate frontier orbitals. However, there also exist many systems in which the electronic structure of an active site, e.g., *d*-band center, dominates. This can be illustrated by SHAP analysis of Pt-terminated Pt_3_Ru_1/2_Ir_1/2_ and Pt_3_Ag surfaces relative to pure Pt, as shown in Supplementary Fig. 24. The effect of adsorbate resonance energies on chemical bonding can be illustrated through a graphic solution to the Newns–Anderson model in Fig. 5b. The upper intersect of an adsorbate line with the Hilbert-transformed chemisorption function represents the adsorbate-metal antibonding state. In accordance with the DFT-calculated density of states projected onto the N 2*p*_*z*_ orbital, an upshift (downshift) in the adsorbate resonance energy results in higher (lower) lying antibonding states pinning across the Fermi level, thus influencing the occupancy of those states and adsorption energies. Consequently, the Co plays an important role in tailoring the adsorbate resonance energies of frontier orbitals, pushing the site motif toward the top of the activity volcano. A global SHAP analysis for N_b_ adsorption models in Fig. 5c further consolidates a pivotal role of adsorbate resonance energies in chemical bonding across a diverse range of ternary Pt alloy surfaces.

## Discussion

Theory integration into deep learning provides an opportunity to further advance the theory of chemisorption at metal surfaces by learning from data. Instead of assuming invariant adsorbate resonance energies of frontier orbitals upon a perturbation of a *d*-metal site, often realized through strain and/or ligand engineering, feature extraction algorithms by convolutional operations automatically learn the tunability of such a resonance state in multimetallic systems. The physical insights into chemical bonding at metal surfaces from interpretable deep learning have important consequences in rational design of improved catalytic systems, by manipulating a broad range of site attributes, e.g., electronegativity, number of *d*-electrons, *d*-orbital radius, and atomic size. With adsorbate resonance energies as tunable factors, many strategies can be envisioned, e.g., alkali metal promoters^45^ and electron tunneling by forming a p-n junction diode^46^. In fact, alkali promoters are often used in heterogeneous catalysis for modifying the catalytic activity of metal nanoparticles. In addition to the electrostatic and direct chemical bonding effects proposed previously^47,48^, the shift of the adsorbate resonance energies due to the electron donation onto the substrate can contribute to the activation of molecular bonds, e.g., dinitrogen (N_2_) in ammonia synthesis. Furthermore, theoretical advances attained here would allow us to go beyond binding sites for improved catalytic performance, such as by modifying the secondary coordination sphere^49,50^ of the adsorption site as a strategy to tailor adsorbate resonance energies. Having been recently used to rationalize the nonlinear scaling of *C and *O adsorption energies on metal surfaces^51,52^, adsorbate resonance energies can be leveraged to target a particular orbital channel or adsorbate for selective chemistries by breaking linear adsorption-energy scaling relations that are largely limiting the attainable catalytic performance in heterogeneous catalysis^53,54^.

In summary, we have established an experimentally validated, interpretable design of Ir-free trimetallic electrocatalysts with {100}-type site motifs for ammonia oxidation. While the screening of high-performance electrocatalysts can be a daunting task, we showed that graph neural networks trained on ab initio data can significantly speed up the process by concurrently predicting site reactivity, surface stability, and catalyst synthesizability descriptors. Experimental results support that Pt_3_Ru–M (M: Co, Ni, or Fe) alloy nanocubes are more active than the state-of-the-art Pt and its bimetallic alloy electrocatalysts, while retaining a relatively superior stability. More importantly, model interpretations enabled by theory integration in deep learning uncover an important physical factor, i.e., adsorbate resonance energies of frontier orbitals, in governing the reactivity of *d*-metal alloy surfaces, and shed light on strategies for designing high-performance catalytic systems beyond the *d*-band center metric of binding sites.

## Methods

### DFT calculations

Density Functional theory (DFT) calculations were performed using Vienna Ab initio Simulation Package (VASP)^55^ with projector augmented wave pseudopotentials. Non-spin-polarized calculations were performed for all systems unless it contained Mn, Ni, Fe or Co in which case spin-polarized calculations were done. For spin-polarized calculation the initial magnetic moment was set to 3, 3, 1 and 2 ${\mu }_{B}$ for Mn, Fe, Ni and Co, respectively, and 0 ${{{{{{\rm{\mu }}}}}}}_{{{{{{\rm{B}}}}}}}$ for any other elements. The exchange-correlation was approximated at the GGA level using the RPBE functional^56^. A plane wave energy cutoff of 450 eV was used and all surfaces were modeled using (2 × 2) supercells with 4 layers. Scaling relations were developed via calculations on the fcc(100) facet of Pt, Ir, Ni, Rh, Pd Au and Ag using lattice constants (in Å) of 3.99, 3.89, 3.55, 3.86, 3.98, 4.20 and 4.21 respectively. For adsorption systems, the bottom two layers were fixed during geometry optimization while the top two layers and adsorbates were allowed to relax until the forces were less than 0.05 eV/Å. A vacuum of 15 Å was used such that there was 30 Å vacuum between two periodic slab images. To calculate energies for gas-phase species the molecule was placed in a 21 Å × 22 Å × 23 Å unit cell. The electronic convergence criteria for all calculations was 10^−5 ^eV. A Monkhorst-Pack mesh of 6 × 6 × 1 was used to sample the Brillouin zone for surfaces and adsorbates while for molecules only the gamma point was sampled. The Methfessel-paxton smearing scheme was used with a smearing parameter of 0.1 eV and precision was set to ‘accurate’. After geometry optimization, the electronic energies are extrapolated to *k*_B_*T* = 0 eV. Density of states were calculated after the geometry optimization via a single point calculation on a denser Monkhorst-Pack mesh of 12 × 12 × 1. Solvation was considered for both slabs and adsorbates using implicit solvation via VASPsol^57,58^, with the dielectric constant and Debye length set to 78.4 and 3 Å, respectively. The surface tension parameter (tau) was set to 0 in order to get rid of electrostatic potential fluctuations in the vacuum. Transition states were located through the use of the nudged elastic band method (NEB)^59^ with 8 images using the same force criteria as geometry optimizations. For the dehydrogenation barriers the initial states always consisted of a coadsorbed *OH and four water molecules and in the final state the *OH has deprotonated the adsorbate (*NH_2_ or *NH) to form a fifth water molecule. The dehydrogenation barriers were converted to electrochemical dehydrogenation barriers using the approach by Nie et al.^30,60,61^, in which the thermochemical activation barrier is approximated to be the same with the electrochemical barrier at the potential of *OH adsorption. The barrier at other potentials is calculated via the Butler-Volmer formalism of the activation free energy using a symmetry factor of 0.5. In order to model the electrode/electrolyte interface, grand-canonical DFT calculations were performed on the Pt(100) surface at 0.3 V_RHE_. Here we briefly describe the key concepts however further details can be seen in our previous work^3^. The electrode potential of a system can be calculated based on the difference in the system work function ($\phi$) and the SHE work function (4.43 eV). If the vacuum potential is at zero then the system work function is related to the fermi level (${E}_{{{{{{{\rm{fermi}}}}}}}}=-\phi$). Thus, a specific potential can be maintained by changing the system’s fermi level (and thus the work function) to the necessary value. This change in fermi level is done by optimizing the number of electrons till the correct work function is found. This optimization of the number of electrons with respect to the potential was done via Newton’s method and we generally found convergence to the required potential in 4-5 iterations.

To convert the DFT energies into free energies we added the zero-point energy (ZPE) and entropic contributions which are shown in Supplementary Table 1. The ZPE and entropic contributions were considered using the harmonic oscillator approximation. For gas-phase species, the translational and rotational contributions to internal energy and entropy were considered via statistical mechanics. For adsorbates, all degrees of freedom were treated as vibrational. All corrections are calculated by setting the temperature to 298 K. The free energy of formation for *NH_2_, *NH, *N and the dehydrogenation transition states were calculated with reference to a *OH covered surface (${\theta }_{{{{{{{\rm{OH}}}}}}}}$ = 0.25), NH_3(g)_ and proton–electron pairs. For *HNNH, *NNH, *NN and the dimerization transition states, the free energy of formation was calculated with reference to a clean slab, NH_3(g)_ and proton–electron pairs. The final activation free energy for dimerization was calculated from the difference in dimerization transition state free energy and the free energy for the initial states (with *OH) far apart. The free energy of the proton–electron pair was calculated via the computational hydrogen electrode^62^, where it was set to half the free energy of hydrogen (H_2_) and a potential dependence (on the RHE scale). Finally, an additional adsorbate dependent correction was added to its formation free energy, to account for the electrode/electrolyte interface. This correction was calculated from grand-canonical DFT calculations on the Pt(100) surface. Specifically, on Pt(100) the adsorbate and transition state formation free energies were calculated under standard DFT and grand-canonical DFT at *U* = 0.3 V_RHE_, and the difference between these two formation free energies corresponds to the correction term. It should be noted that for the grand-canonical calculations an additional term (${-E}_{{{{{{{\rm{fermi}}}}}}}}\times {N}_{{{{{{{\rm{electrons}}}}}}}}$) was added when calculating the intermediate Gibbs free energies which accounts for the electrons added/removed (${N}_{{{{{{{\rm{electrons}}}}}}}}$) to achieve the required potential.

### Microkinetic modeling

The activity maps were developed using CATMAP^63^ at an operating potential of 0.3 V_RHE_ with the ammonia activity approximated to be 0.1 (*c*_NH3_ = 0.1 M). Adsorbate-adsorbate interactions were included for *NH_2_ using a first order interaction model where the adsorbate interaction function was modeled via a smooth piecewise linear function. The *NH_2_ adsorbate interaction parameter was calculated on Pt(100) by plotting the *NH_2_ formation free energies as a function of coverage as shown in Supplementary Figure 3a. Within the microkinetic model, we allow for the reactions to occur on either the bridge or hollow sites. The deprotonation barriers of *HNNH and *NNH were set to a value of 0.25 eV since it was expected that these steps would never be rate limiting. For the activity map two descriptors were used which were the nitrogen bridge and hollow binding energies (${\varDelta E}_{N}$), which were both calculated with respect to a gas phase nitrogen atom in a box. Scaling relations between the formation free energies (at *U* = 0.3 V_RHE_) of the adsorbates or transition state and these two descriptors are shown in Supplementary Figure 2.

### Graph neural networks

All machine learning models in this work were based on graph neural networks (GNN). The GNN consists of two parts: convolutional network (CNN) and fully-connected neural network (FCNN). The convolutional network extracted high-level features from the graph feature representation of adsorbate-substrate systems, and the fully-connected neural network mapped high-level features to properties of interest or governing factors in the theory module. Five different graph neural networks were trained including two classification GNNs to predict the likelihood of surface reconstruction for bridge and hollow *N structures, and three regression models. One of the regression models was built to predict the formation energies of the *OH covered alloy surfaces, and two more regression models were built to predict the hollow and bridge *N binding energies. For the binding energy predictions, we used the TinNet that integrates GNNs with a theory module to allow for model interpretability. TinNet models used in this study adopted the Newns-Anderson-type model Hamiltonians in the theory module.

GNNs have 5 hyperparameters that require optimization, including the number of layers and neurons within the layer of convolutional network and fully-connected neural network and the learning rate. The Bayesian optimization algorithm in the Ray Tune package was used for hyperparameter optimization, which is a Python library for hyperparameter tuning at any scale^64,65^. The regular 10-fold cross-validation method was adopted in the hyperparameter optimization and a total of 10 models were trained for each hyperparameters set. For each training, we used an 81/9/10 training/validation/test split. The validation set was used for early stopping to avoid overfitting the training set. The test set was used to evaluate the performance of the trained models. The hyperparameters set with the smallest average loss is the optimal hyperparameters set, which will be used in subsequent model training.

The nested 10-fold cross-validation was performed to avoid overfitting. The database was divided into 10 folds, of which one fold was used for validation, 1 fold was used for test, and the rest was used for training, where the validation and test can be the same fold and a total of 100 models were trained. The 90 models whose test set was not equal to the validation set were used to study the in-sample prediction performance of the models. Other 10 final models were used for the design space of ternary Pt alloys to demonstrate the out-of-sample predicting performance of models and to suggest candidates worthy of performing DFT calculations. Since this study focused on Pt-based catalysts, the pure Pt system was always included in the training set. The AdamW^66^ was used as the optimizer to update weights and biases of models. To avoid getting stuck in local minima, the training set was split into multiple mini-batches where the size of each mini-batch was 64. The negative log likelihood (NLL) and the mean squared error (MSE) were used as loss functions for classification and regression problems, respectively. For TinNet models, the loss function contained not only the adsorption energy, but also the low-order *d*-band moments of the active site and projected density of states onto N2p orbitals.

### SHAP analysis

Shapley values were computed by a model-independent kernel as implemented in the Shapley additive explanations (SHAP) (version 0.36.0) package to explain the governing electronic factors extracted by TinNet. The Shapley value concept was first established to measure a player’s importance within a team^67^. With this idea, players would get a share of the overall benefit or reward based on how important was the role they played in the game’s result. Within the context of TinNet predicted binding energies, Shapley values can be used to find how the electronic factors learnt from TinNet, govern the binding energy for any given system. Shapley values estimate the feature importance, in terms of how much it impacts the final output (magnitude of SHAP values) as well as the direction in which it can influence the final output (sign of SHAP value). Specifically, for binding energy predictions, positive values indicate that the electronic feature contributed to increasing the adsorption energy positively, i.e., weakening of the bond whereas negative sign shows a contribution to strengthening the bond. Shapley values are computed as:

1 $${{{{{{\rm{\varphi }}}}}}}_{j}({{{{{{\rm{val}}}}}}})=\mathop{\sum}\limits_{S\subset \{1,\ldots,p\}{{\backslash }}\{j\}}\frac{\left|S\right|!\left(p-\left|S\right|-1\right)!}{p!}({{{{{{\rm{val}}}}}}}\left(S\cup \{j\}\right)-{{{{{{\rm{val}}}}}}}\left(S\right))$$

The function val(*S*) estimates the overall contribution of a given set of features (val(*M*) = *f*(*x*_*i*_)), and the sum is across all potential subsets where feature *i* is not included.

### Catalysts synthesis

GO was synthesized according to an improved Hummers method^68^. A shape-directing agent-assisted reduction method was used to prepare rGO-supported Pt, Pt_3_M or Pt_3_Ru_1/2_*M*_1/2_ (*M* = Fe, Co or Ni) nanocubes, in which the metal mass percentage in all samples was controlled to be 20 wt%. Herein, the synthesis of rGO-supported Pt_3_Ru_1/2_Co_1/2_ nanocubes was used as an example to simply summarize the synthesis details. Typically, 0.05 mmol of potassium tetrachloroplatinate (II) [K_2_PtCl_4_] (≥ 45% Pt, Aladdin), ruthenium chloride (III) [RuCl_3_] (45-55% Ru, Aladdin) and cobalt chloride (II) [CoCl_2_] (97% Co, Aladdin), as well as 34 mg of GO powder were added into a round-bottom flask that contains 20 mL of ethylene glycol (EG, Sinopharm Chemicals). The solution was then stirred for 30 min at room temperature to form a homogeneous mixture. Afterward, 0.75 mmol of tetramethylammonium bromide (C_4_H_12_BrN, Aladdin) was subjected into the mixture and sonicated for 5 min, then the flask was transferred into an oil bath. Thereafter, the reaction mixture was heated to 180 °C with stirring and condensing reflux for 30 min. After cooling down to room temperature, the final products were washed with deionized water at least three times and then freeze-dried at –50 °C for 24 h. Additionally, the iron and nickel precursors used are iron chloride (III) [FeCl_3_] (≥ 99.9% Fe, Aladdin) and nickel chloride (II) [NiCl_2_] (99.9% Ni, Aladdin), respectively.

### Physical characterization

The overall particle size and distribution of the catalysts’ morphologies were characterized by scanning electron microscopy on JSM-7800 F microscope at a working voltage of 20 kV. The crystal phases in catalyst samples were analyzed by using powder XRD on a Philips 1730 diffractometer with Cu K_α_ radiation (*λ* = 1.5406 Å). XPS was conducted using a Thermo ESCALAB 250 spectrophotometer equipped with an Al K_α_ line source. Atomic-resolution HAADF images of atomically dispersed Pt sites in the ternary alloy nanocubes were captured in a FEI Theims Z STEM operated at 300 kV. TEM and HR-TEM images were performed on Tecnai G2-F30 equipped with an EDS. The ICP-MS was tested on NexION 300X Spectrometer to determine the metal content in the catalyst. Ultraviolet photoemission spectroscopy (UPS) were performed on an ESCALAB 250Xi electron spectrometer (Thermo Fisher Scientific) using a monochromatic Al Kα source (300 W).

### Electrochemical measurements

For the ink preparation, the catalyst powder (2 mg, e.g., Pt_3_Ru_1/2_Co_1/2_/rGO) was mixed with isopropanol (994 μL, Sinopharm Chemicals) and 5 wt% Nafion solution (6 μL, Sigma-Aldrich) under ultrasonication for about 30 min, and then the final ink was ready to use. Afterward, a 10 μL of the prepared ink was drop-cast on the glassy carbon electrode and dried in air to yield a thin-film electrode, which was ready for the electrochemical test. For all the studied catalysts, the Pt loading was fixed at about 8 μg cm^−2^ for each tested electrode, estimated based on the overall Pt ratio within the catalyst (determined by ICP-MS). The AOR performance of the catalysts was measured by the rotating disk electrode (RDE, 5.0 mm in diameter) techniques with a CHI Electrochemical Station (Model 760E) in a three-electrode electrochemical cell. KCl-saturated Ag/AgCl electrode and a graphite rod were used as the reference and counter electrodes, respectively. Cyclic voltammetry measurements were firstly conducted in an Ar-saturated 1.0 M KOH between 0.05 and 1.1 V vs RHE at a scan rate of 100 mV s^−1^ to activate the catalysts. The activated electrode was then measured in an Ar-saturated 1.0 M KOH + 0.1 M NH_3_ solution at room temperature and an RDE rotation rate of 900 rpm in the same potential window. Note that the NH_3_ solutions were prepared by mixing NH_3_ solution (25 − 28%, RHAWN) with DI water and KOH. Chronoamperometry (CA) measurements were also conducted at 0.55 vs RHE for 1,800 s and a rotation rate of 900 rpm to further evaluate the AOR activity changes. During each AOR measurement, a fresh electrode was prepared and used to standardize the testing procedure.

## Supplementary information

Supplementary Information

Peer review file

## Data Availability

Currently, all source data for microkinetic modeling and machine learning that were used in this study are available from the GitHub repository: https://github.com/hlxin/aor.
