# Author Correction: Interpretable design of Ir-free trimetallic electrocatalysts for ammonia oxidation with graph neural networks

**DOI:** 10.1038/s41467-023-36746-z

**Published:** 2023-02-20

**Authors:** Hemanth Somarajan Pillai, Yi Li, Shih-Han Wang, Noushin Omidvar, Qingmin Mu, Luke E. K. Achenie, Frank Abild-Pedersen, Juan Yang, Gang Wu, Hongliang Xin

**Affiliations:** 1 Department of Chemical Engineering, Virginia Polytechnic Institute and State University, Blacksburg, VA USA; 2 School of Materials Science and Engineering, Jiangsu University, Zhenjiang, Jiangsu China; 3 SUNCAT Center for Interface Science and Catalysis, SLAC National Accelerator Laboratory, Menlo Park, CA USA; 4 Department of Chemical and Biological Engineering, University at Buffalo, The State University of New York, Buffalo, NY USA

**Keywords:** Materials for energy and catalysis, Catalytic mechanisms, Electrocatalysis, Theoretical chemistry

Correction to: *Nature Communications* 10.1038/s41467-023-36322-5, published online 11 February 2023

The original version of the published article omitted acknowledgement to the U.S. DOE. The following statement is now added in the Acknowledgement section in both HTML and the PDF versions of the article:

“F.A-P. would like to acknowledge support from the U.S. DOE, Office of Science, Office of Basic Energy Sciences, Chemical Sciences, Geosciences, and Biosciences Division, Catalysis Science Program to the SUNCAT Center for Interface Science and Catalysis.”

