# Supplementary material for: Interpretable design of Ir-free trimetallic electrocatalysts for ammonia oxidation with graph neural networks
Source: Nat Commun. 2023 Feb 11;14:792. doi: 10.1038/s41467-023-36322-5 (PMC9922329; doi:10.1038/s41467-023-36322-5)
Supplement: Supplementary file 1 — Supplementary Information [file 41467_2023_36322_MOESM1_ESM.docx]

**Supplementary Information for: Interpretable Design of Ir-free Trimetallic Electrocatalysts for Ammonia Oxidation with Graph Neural Networks**

**Hemanth Somarajan Pillai^1^, Yi Li^2,*^, Shih-Han Wang^1^, Noushin Omidvar^1^, Qingmin Mu^1^, Luke E. K. Achenie^1^, Frank Abild-Pedersen^3^, Juan Yang^2^, Gang Wu^4,*^, and Hongliang Xin^1,*^**

*^1^Department of Chemical Engineering, Virginia Polytechnic Institute and State University, Blacksburg, VA, USA*

*^2^School of Materials Science and Engineering, Jiangsu University, Zhenjiang, Jiangsu, China*

*^3^SUNCAT Center for Interface Science and Catalysis, SLAC National Accelerator Laboratory, Menlo Park, CA, USA*

*^4^Department of Chemical and Biological Engineering, University at Buffalo, The State University of New York, Buffalo, NY, USA*

**Supplementary Discussion**

**Physical parameters within TinNet.** The physical parameters represented by the neural state variables include:

Properties of the site atoms, two for the bridge site and four for the hollow site.

- *d*-band center ($\epsilon_{d})$
- *d*-band width ($w_{d})$

Properties of the adsorbate-substrate interaction of frontier orbitals, i.e., *p_x_*, *p_y_*, and *p_z_.*

- adsorbate resonance energy ($\epsilon_{p{}_{i}}^{a}$)
- interatomic coupling coefficient ($\beta{{}_{p}}_{i}$)
- height of the rectangular *sp*-band chemisorption function ($\Delta_{p_{i}}^{0}$)

We also have the orbital overlap coefficient ($\alpha$) and the sp contribution ($E_{sp}$) that are orbital-independent and global. The values for $\alpha$ and $E_{sp}$ for each site are calculated from *Bayeschem*^1^.

To summarize, for the bridge site *N_b_, 13 physical parameters were trained (2 $\epsilon_{d}$, 2 $w_{d}$, 3 $\epsilon_{p{}_{i}}^{a}$, 3 $\beta{{}_{p}}_{i}$, and 3 $\Delta_{p_{i}}^{0}$). Alpha and E_sp_ were global constants set to 0.0441 eV^-1^ and -3.343 eV respectively which were calculated via *Bayeschem* ^1^.

For the hollow site, 17 physical parameters were trained (4 $\epsilon_{d}$, 4 $w_{d}$, 3 $\epsilon_{p{}_{i}}^{a}$, 3 $\beta{{}_{p}}_{i}$, and 3 $\Delta_{p_{i}}^{0}$). Alpha and E_sp_ were set to global constants of 0.0585 eV^-1^ and -4.24 eV respectively.

Since the $w_{d}$, $\beta{{}_{p}}_{i}$, and $\Delta_{p_{i}}^{0}$ must be positive values, the softplus activation function was used to constraint the range of neural state variables at the output layer. The $\epsilon_{d}$and $\epsilon_{p{}_{i}}^{a}$ can be either positive or negative, for which the linear activation function was used.

**Input graph features.** The input graph features contain atomic features for each atom and bond features between each atom and its neighbours. As in the ref^2^ atomic features contain 9 properties, including the group number, period number, electronegativity, covalent radius, valence electrons, first ionization energy, electron affinity, element block on the periodic table, and atomic volume. Each property was characterized by a number of categories. For example, the element block has four categories, i.e., s, p, d and f blocks. For each atom, a set of boolean values were used to indicate the element category, i.e., one-hot encoding. With this approach, the block feature of a gold atom is [0 0 1 0], which represents the atom of interest belonging to the d-block on the periodic table. To capture bond information of an atom with its neighbors, a Gaussian basis function with 41 filters (from 0 to 8 Å with 0.2 Å interval) is used to expand the distance as a vector. The stack of atomic features and bond features forms the graph of a system.

Graph feature representations can be very costly in terms of memory. The GPU nodes used in this study have P100, V100 or T4 GPUs with 12-16 GB memory per GPU and can process up to 10,000 - 20,000 images simultaneously. Our training dataset contains a few hundreds of images. So we didn't run into the out-of-memory issue. We just need a single GPU card to complete the model training for a given hyperparameter set and data splitting within a few hours. Distributed data parallel training can be used with multiple GPU cards for large datasets. This is a way to overcome the out-of-memory issue if encountered and can speed up the training process.

**Hyperparameter optimization.** For hyperparameter optimization, we adopted the regular 10-fold cross validation approach. For each hyperparameter set, a total of 10 models were trained. Before each training, we split off 10% of the whole dataset for the test, which was never used during training. During the training process, we randomly took 10% of the training dataset as the validation set in order to stop training early to prevent overfitting. The 10% of the training dataset was equal to 9% of the entire dataset. Hence, for each training, 81% of the dataset was used for training, 9% for validation, and 10% for test.

In the active learning phase, we adopted the nested 10-fold cross validation approach^3^. The dataset was divided into 10 folds with 1 fold for test, 1 fold for validation, and all the rest for training. For a given dataset, a total of 100 models were trained with the optimized hyperparameter set. Since the test fold and validation fold can be the same one, the training/validation/test split is either 80/10/10 or 90/10/0. The 90 models among 100 models with the 80/10/10 split were used to evaluate the in-sample model performance, i.e., parity plots in the manuscript. The other 10 models with 90/10/0 split were used to evaluate out-of-sample model performance, i.e., screening materials in the design space.

**Supplementary Table 1| Free energy corrections to adsorbates and gas phase species.** For gas phase species, corrections were calculated by using statistical mechanics and considering the vibrations, rotational and translational degrees of freedom. The pressure of NH_3_(g), H_2_(g) were set to 1 bar. While the pressure of H_2_O(g) was set to the water vapor pressure (0.035 bar) at 298 K. For adsorbates, all degrees of freedom were treated as vibrational via the simple harmonic oscillator. An additional constant potential correction was added which accounts for the effect of the electrochemical bias at the electrode-electrolyte interface. The correction was calculated from grand-canonical DFT for all adsorbates on a Pt(100) surface at 0.3 V vs. RHE. Details of the grand-canonical DFT calculations are reported in the Methods section. All values are in units of eV.

| Adsorbate | ZPE | TS | Constant potential |
| --- | --- | --- | --- |
| *NH_2_ | 0.73 | 0.06 | 0.10 |
| *NH_b_ | 0.36 | 0.07 | -0.01 |
| *NH_h_ | 0.36 | 0.07 | 0.11 |
| *N_b_ | 0.10 | 0.04 | -0.10 |
| *N_h_ | 0.10 | 0.04 | -0.09 |
| *NH-NH | 0.72 | 0.14 | 0.24 |
| *NH-N | 0.46 | 0.11 | 0.21 |
| *N-N | 0.20 | 0.08 | -0.10 |
| *HNNH | 0.86 | 0.07 | 0.22 |
| *NNH | 0.52 | 0.07 | 0.24 |
| *NN | 0.19 | 0.07 | 0.03 |
| NH_3_(g) | 0.95 | 0.60 | 0.00 |
| H_2_(g) | 0.27 | 0.40 | 0.00 |
| H_2_O(g) | 0.58 | 0.65 | 0.00 |

**Supplementary Table 2| Hollow and bridge N binding energies (in eV) for all metal surfaces shown in Figure 1b**. The binding energies were calculated with respect to an *OH covered surface ($\theta_{\mathrm{OH}}$ = 0.25) and a gas-phase nitrogen atom.

| **Surfaces** | ${\Delta E}_{*\mathrm{Nb}}$ | ${\Delta E}_{*\mathrm{Nh}}$ |
| --- | --- | --- |
| Pt(100) | -4.08 | -3.68 |
| Ir(100) | -4.75 | -4.53 |
| Rh(100) | -4.51 | -4.82 |
| Pd(100) | -3.44 | -4.17 |
| Au(100) | -1.57 | -0.67 |
| Ag(100) | -0.91 | -1.88 |
| Pt_3_Ir(100) | -4.79 | -4.69 |

**Supplementary Figure 1| Free formation energies of key intermediates on Pt(100) as a function of the electrode potential.** Specifically, coadsorbed *NH_2_ and *OH ($\theta_{\mathrm{OH}}$ = 0.25, $\theta_{NH2}$= 0.25), coadsorbed *NH and *OH ($\theta_{\mathrm{OH}}$ = 0.25, $\theta_{\mathrm{NH}}$= 0.25), coadsorbed *NH_2_ and *N_2_H_4_ ($\theta_{NH2}$ = 0.625, $\theta_{N2H4}$= 0.0625) and 0.75 ML of *NH_2_ are shown. While the dimerization of *NH_2_ to form *N_2_H_4_ is thermoneutral at 0.75 ML *NH_2_, the *OH coadsorbed surfaces are more favorable than the 0.75 ML *NH_2_ surface. It suggests that under operating conditions it is unlikely that a high coverage of *NH_2_ forms, which prevents the formation of hydrazine (*N_2_H_4_) via *NH_2_ dimerization.

**Supplementary Figure 2| Linear adsorption-energy scaling relations.** Free formation energies of adsorbates (**a** and **b**) and transition states (**c**) as a function of either the bridge (${\Delta E}_{*\mathrm{Nb}}$) or hollow N (${\Delta E}_{*\mathrm{Nh}}$) binding energies. The equations for each scaling relation are shown within the plots. Free formation energies were calculated with respect to NH_3_(g), H_2_(g) and an *OH covered surface ($\theta_{\mathrm{OH}}$ = 0.25). The transition states scaling relations for dimerization (N-NH, N-N and NH-NH) and dehydrogenation (N-H and NH-H) are shown in **c**. The bridge and hollow N binding energies were calculated with respect to a gas-phase nitrogen atom and an *OH covered surface ($\theta_{\mathrm{OH}}$ = 0.25).

**Supplementary Figure 3| The average *NH_2_ and *NH formation free energy as a function of the *NH_2_ and *NH coverage on Pt(100)** **and the AOR activity map at 0.3 V with the influence of *NH_2_ and *NH coverage dependent energetics.** (**a**) The average *NH_2_ and *NH formation free energy was calculated via DFT at *NH_2_ and *NH coverages of 0.25, 0.5 and 0.75. Subsequently, a linear piecewise relationship was fit and then incorporated into the microkinetic model via CATMAP, the interaction parameters for both adsorbates are reported ($\varepsilon_{NH_{2}}$= 1.5 and $\varepsilon_{NH}$= 1.07). (**b**) AOR activity map at 0.3 V vs. RHE and 298 K with the inclusion of *NH_2_ and *NH coverage-dependent energetics.

**Supplementary Table 3| Effect of compressive strain and metal substitution beyond second layer on the *N binding energy (in eV)**. *N Binding energies were calculated on Pt_3_Ru and Pt_3_Ru systems where the cobalt atom replaced an atom in the third or fourth layer. It can be seen that third- and fourth-layer substitutions have <0.1 eV influence on the binding energies. Binding energies on a Pt_3_Ru system with the Pt_3_Ru_1/2_Co_1/2_ lattice constant were also calculated (-0.9 %), to show the negligible effect of strain. Energies are calculated with respect to an *OH covered surface ($\theta_{\mathrm{OH}}$ = 0.25) and a gas-phase nitrogen atom.

| **Surfaces** | ${\Delta E}_{*\mathrm{Nb}}$ | ${\Delta E}_{*\mathrm{Nh}}$ |
| --- | --- | --- |
| Pt_3_Ru(100) | -5.11 | -5.27 |
| Pt_3_Ru(100) with a Co in the 3^rd^ layer | -5.03 | -5.22 |
| Pt_3_Ru(100) with a Co in the 4^th^ layer | -5.14 | -5.23 |
| Strained Pt_3_Ru(100) (-0.9 %) | -5.08 | -5.27 |


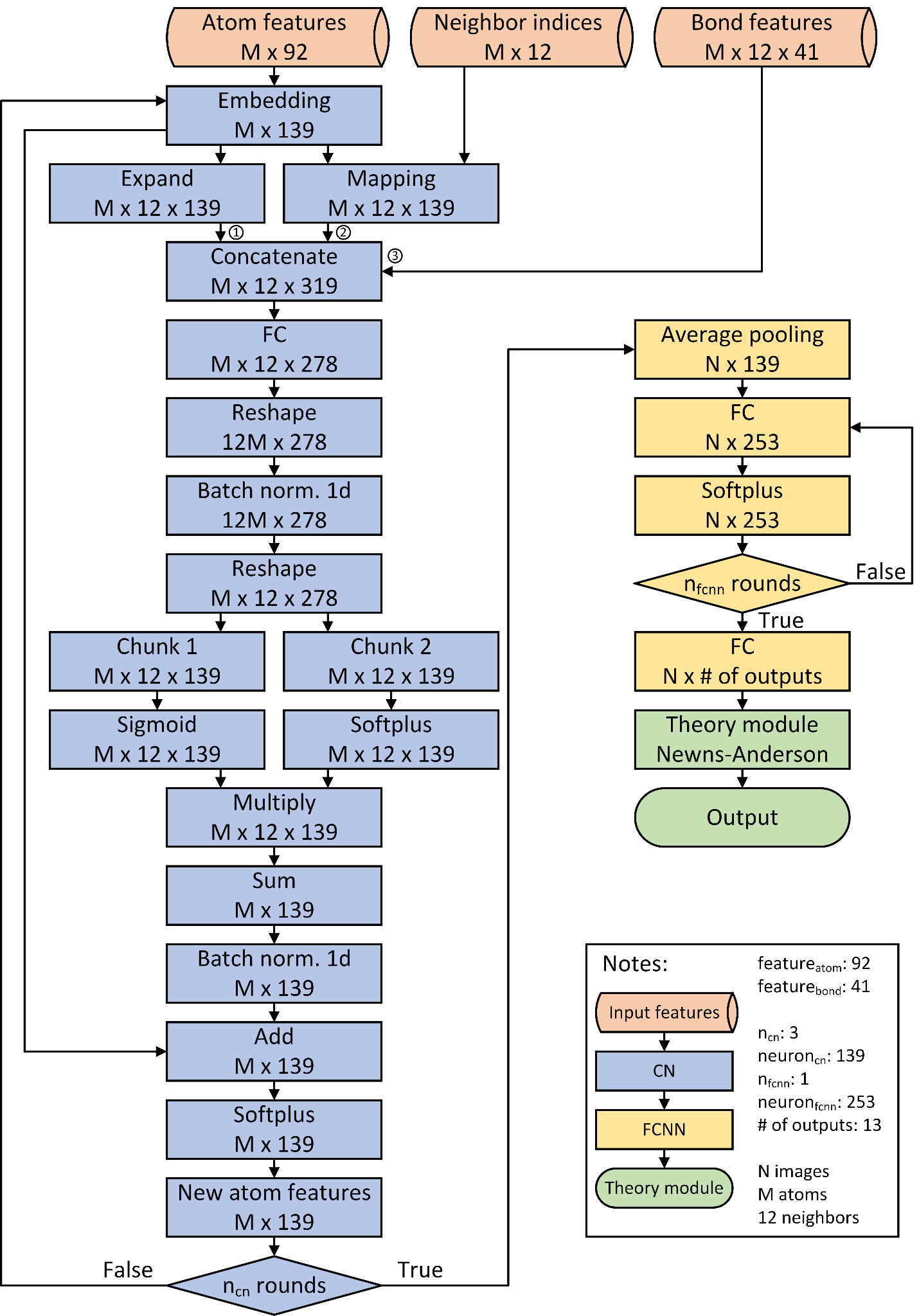


**Supplementary Figure 4| The architecture of graph neural networks.** The GNN consists of a convolutional network (CN) and a fully-connected neural network (FCNN). For the TinNet model an additional theory module (via the News-Anderson Hamiltonian) was integrated into the GNN. The first row in each block represents the name or function of the step. The second row shows the dimensions of the data array. This figure uses hyperparameters of the TinNet model of ${\Delta E}_{*Nb}$ as an example.

**

**

**Supplementary Figure 5| Linear Adsorption energy model.** Parity plot of DFT calculated adsorption energy of *N at the hollow site of {100}-terminated alloy surfaces vs. linear regression models predicted values. Linear regression models adopted six hand-crafted features as input features include Mulliken electronegativity, orbitalwise coordination number of the s-orbital, number of valence electrons, dipole polarizability, lattice constant of an FCC metal phase, and work function.

**

**

**Supplementary Figure 6| Model performance of graph neural networks for predicting the likelihood of surface stability under reaction conditions at different iterations.** Classification models to predict the likelihood of surface stability against reconstruction by the *N at (**a**) the bridge site, and (**b**) the hollow site of {100}-terminated alloy surfaces. The results from all three iterations of the active learning workflow are shown and the performance of the models is measured via the AUC value.

**
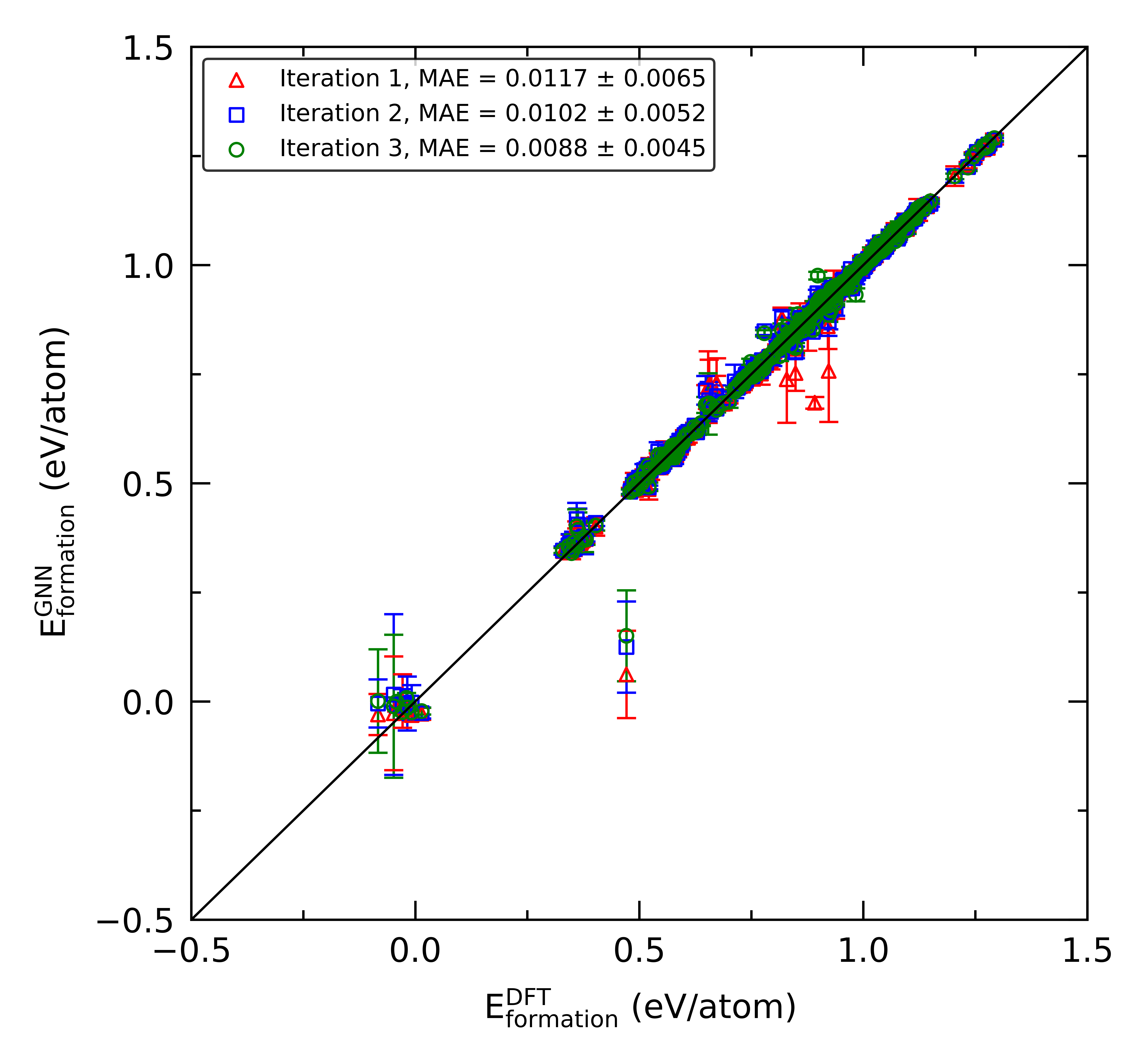
**

**Supplementary Figure 7| GNN models for predicting the formation energies of hydroxylated surfaces.** DFT-calculated vs. GNN-predicted formation energies of *OH covered surface ($\theta_{\mathrm{OH}}$ = 0.25). The reported values are the average from nested 10-fold cross validation while error bars represent the standard deviation from the models. Different markers represent the different iterations of the active learning workflow.





**Supplementary Figure 8| TinNet models for predicting the adsorption energies of *N at hydroxylated surfaces.** DFT-calculated vs. TinNet-predicted *N adsorption energies at the (**a**) bridge site, and (**b**) hollow site of {100}-terminated alloy surfaces. The values represent the average from nested 10-fold cross validation, while error bars represent the standard deviation. Different markers correspond to different iterations of the active learning workflow.








**Supplementary Figure 9| Model development for the electronic structure of the bridge site atoms.** DFT-calculated vs. TinNet-predicted (**a**, **b**) *d*-band center, and (**c**, **d**) *d*-band half-width of two neighboring atoms that bond directly to *N. The TinNet model has the *d*-band center and *d*-band width as neural state variables for generating the surface density of states. Error bars display the standard deviation from the 10-fold cross validation.














**Supplementary Figure 10| Model development for the electronic structure of the hollow site atoms.** DFT-calculated vs. TinNet-predicted (**a-d**) *d*-band center, and (**e-h**) *d*-band half-width of four neighboring atoms that bond directly to *N. The TinNet model has the *d*-band center and *d*-band width as neural state variables for generating the surface density of states. Error bars display the standard deviation from the 10-fold cross validation.

**Supplementary Table 4| Optimized hyperparameter sets of the various machine learning models.** Hyperparameter sets of GNN and TinNet models, including the number of layers in the convolutional network (n_cn_), the number of neurons within the convolutional network (neuron_CN_), the number of layers in the fully-connected neural network (n_fcnn_) and the number of neurons within the fully connected neural network (neurons_fcnn_) and the learning rate. The total number of parameters is tabulated in the last column. The stability model was a classification model while all other three models were regression.

| **Model** | **n_cn_** | **neuron_cn_** | **n_fcnn_** | **neuron_fcnn_** | **learning rate** | **# of parameters** |
| --- | --- | --- | --- | --- | --- | --- |
| Stability | 3 | 64 | 1 | 128 | 1.0000 $\times$ 10^-2^ | 80,962 |
| $E_{formation}$ | 6 | 237 | 2 | 67 | 9.4057 $\times$ 10^-3^ | 1,518,647 |
| $E_{adsorption}^{bridge}$ | 3 | 139 | 1 | 253 | 6.3265 $\times$ 10^-3^ | 321,031 |
| $E_{adsorption}^{hollow}$ | 5 | 177 | 5 | 33 | 7.2775 $\times$ 10^-4^ | 733,631 |

**Supplementary Table 5| The number of *N bridge and hollow structures used for each model within each iteration and the final design space.** The number of structures is listed for each model and iteration. The number of systems included for surface stability include stable and unstable systems. Only systems which are considered stable are used to develop the adsorption energy models. For the formation energy we only considered the *OH covered structures. The last column lists the entire design space for bridge and hollow *N species which was searched via active learning.

|  | Initial | | Iteration 1 | | Iteration 2 | | Design space | |
| --- | --- | --- | --- | --- | --- | --- | --- | --- |
| Model | Bridge | Hollow | Bridge | Hollow | Bridge | Hollow | Bridge | Hollow |
| Surface stability | 742 | 614 | 817 | 689 | 860 | 733 | 9,000 | 15,000 |
| Adsorption energy | 415 | 330 | 485 | 373 | 510 | 375 | 9,000 | 15,000 |
| Formation energy | 415 | | 485 | | 510 | | 9,000 | |

**
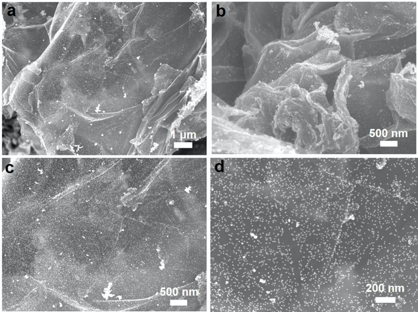
**

**Supplementary Figure 11| Morphology characterization of the rGO supported Pt_3_Ru_1/2_Co_1/2_ cubic nanoparticles by SEM.** (**a, b**) Low-resolution and (**c, d**) high-resolution SEM images, respectively. These results indicate that ternary PtRuCo alloy nanoparticles with cubic-dominant structure were uniformly distributed onto the reduced graphene oxide support.


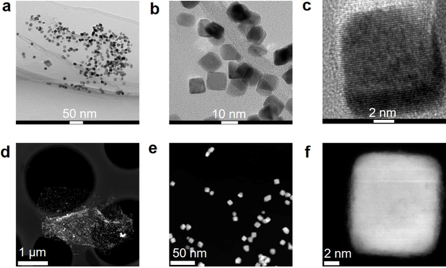


**Supplementary Figure 12| Morphology and structure characterization of the rGO supported Pt_3_Ru_1/2_Co_1/2_ cubic nanoparticles by TEM.** (**a-c**) Bright-field (BF)-STEM images and (**d-f**) HAADF-STEM images, respectively. These results indicate that the reduced graphene oxide supported PtRuCo ternary alloy nanoparticles with cubic-dominant structure were successfully synthesized via a wet-chemistry reduction method.


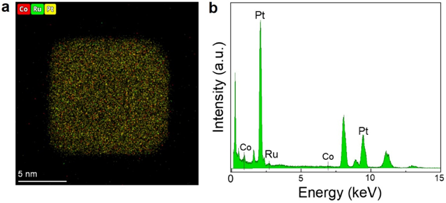


**Supplementary Figure 13| Composition characterization of the rGO supported Pt_3_Ru_1/2_Co_1/2_ cubic nanoparticles by EDS.** (**a**) Integrated maps of Pt, Ru and Co elements and (**b**) the corresponding EDS curve. The results indicate that the stoichiometric Pt/Ru/Co ratio was roughly 3/0.5/0.5.


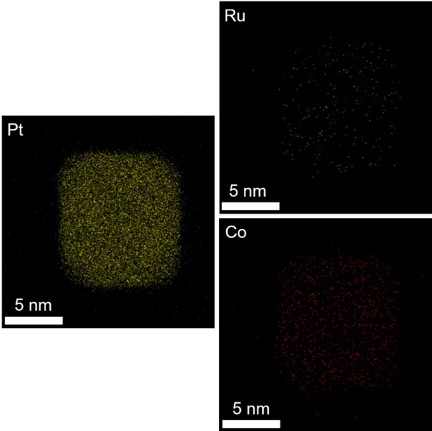


**Supplementary Figure 14| EDS mapping of single PtRuCo alloy nanoparticle.** The figures show different atom columns of Pt (in yellow), Ru (in green) and Co (in red), respectively, indicating the successful introduction of Ru and Co atoms into Pt matrix.

**
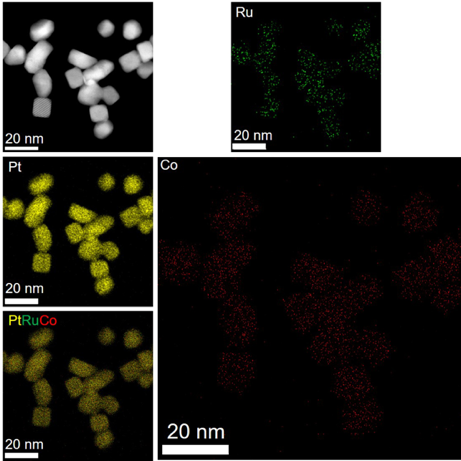
**

**Supplementary Figure 15| HAADF-STEM image and the corresponding EDS mapping of PtRuCo alloy nanoparticles.** The figures show different atom columns of Pt (in yellow), Ru (in green) and Co (in red), respectively, as well as their overlay, which suggest the successful alloying of Ru and Co atoms into Pt matrix.


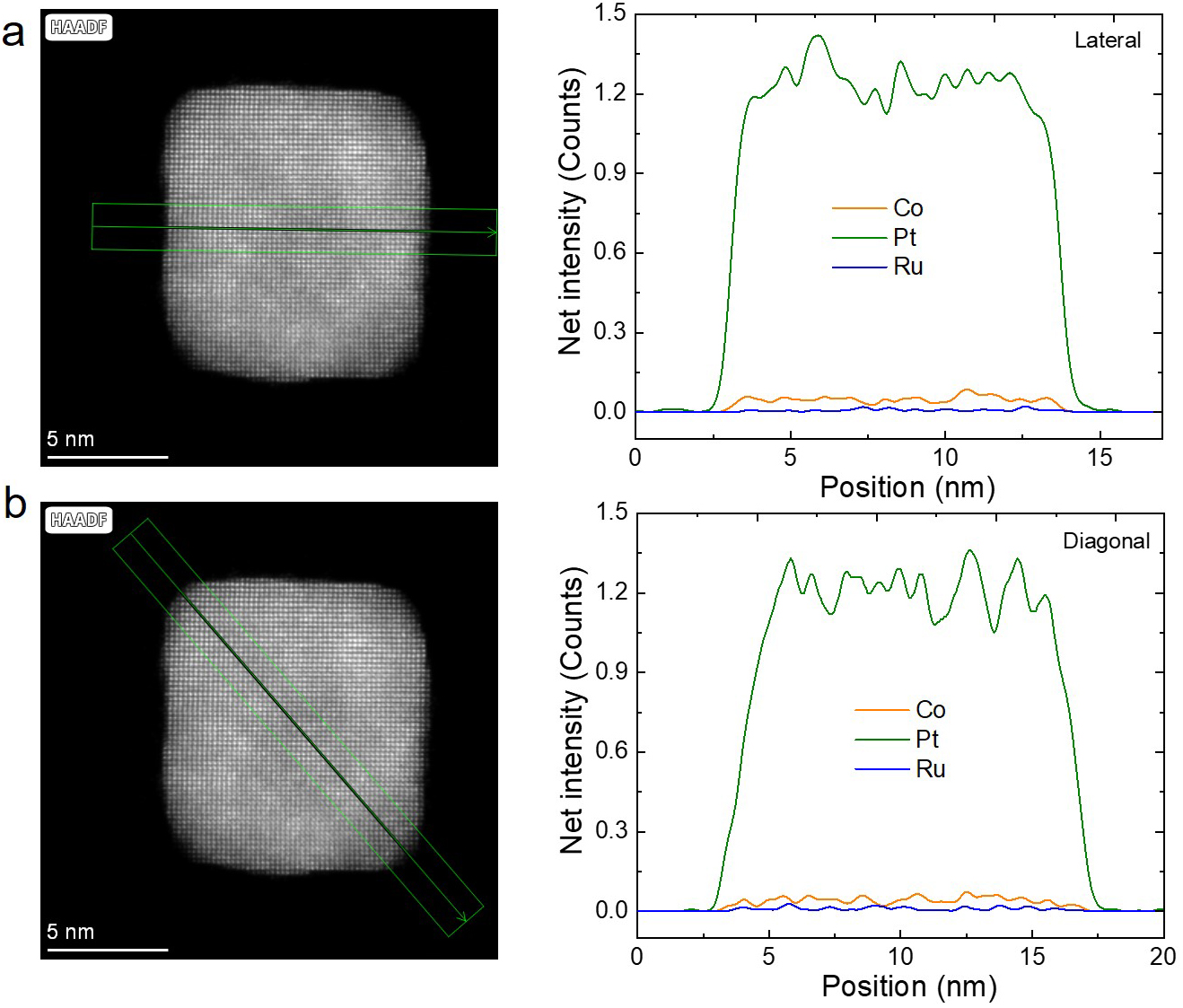


**Supplementary Figure 16| HAADF-STEM image and the corresponding EDS line profiles across Pt_3_Ru_1/2_Co_1/2_ nanoparticles.** In (**a**) lateral and (**b**) diagonal directions.

**
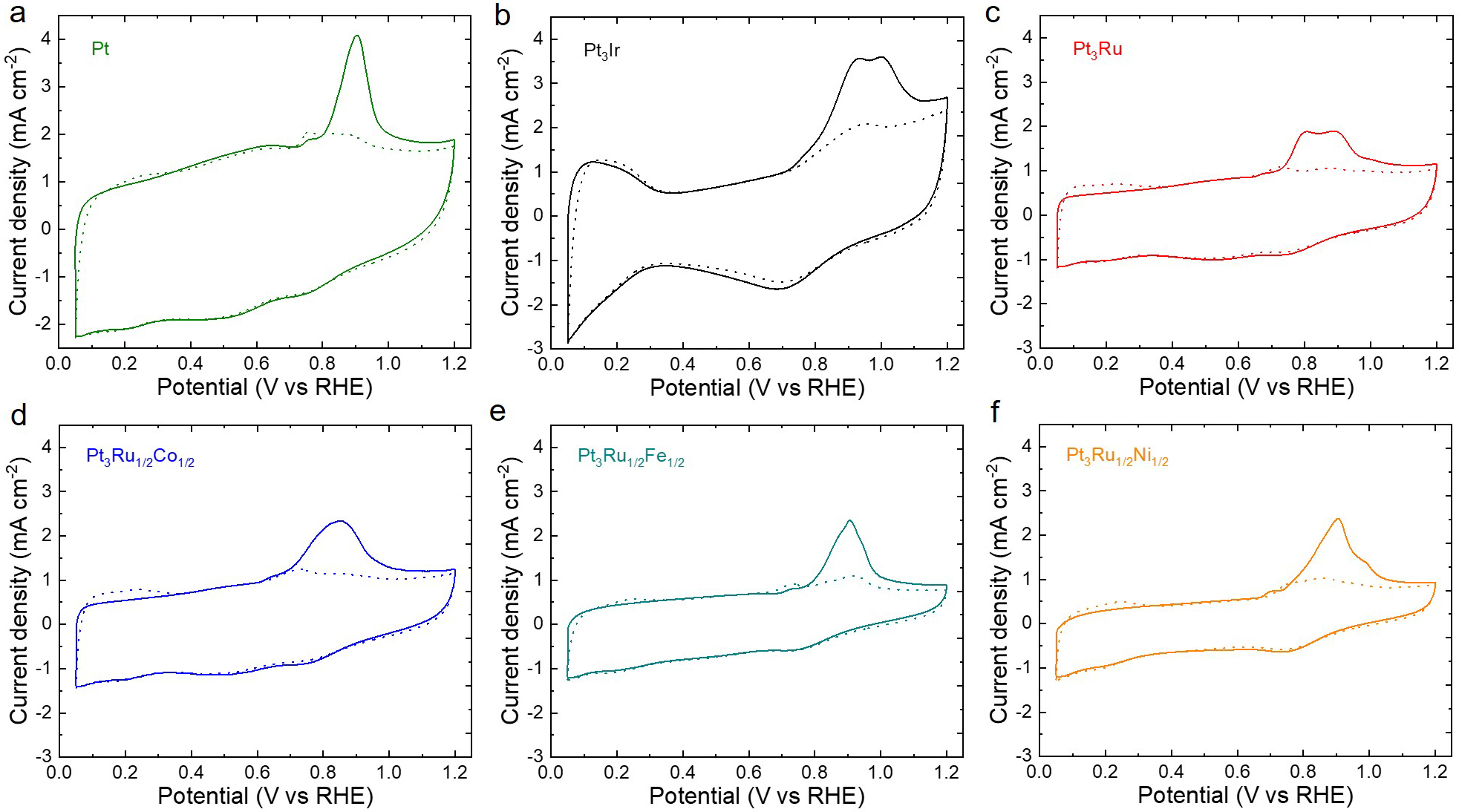
**

**Supplementary Figure 17| CO stripping measurements.** CO stripping voltammograms in CO-saturated 0.1 M HClO_4_ aqueous electrolytes with a scanning rate of 50 mV s^–1^ for (a) Pt, (b) Pt_3_Ir, (c) Pt_3_Ru, (d) Pt_3_Ru_1/2_Co_1/2_, (e) Pt_3_Ru_1/2_Fe_1/2_ and (f) Pt_3_Ru_1/2_Ni_1/2_.


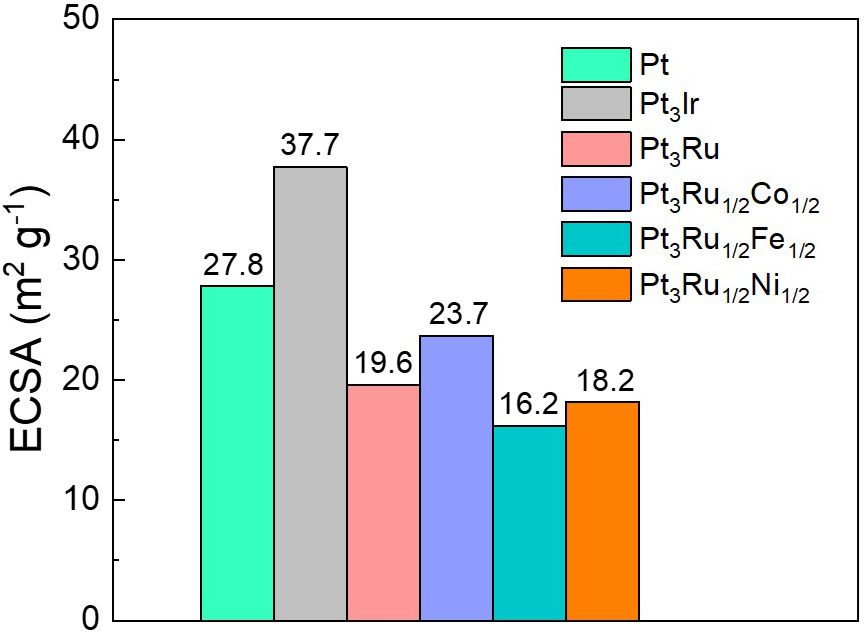


**Supplementary Figure 18| ECSA comparisons for Pt, Pt_3_Ir, Pt_3_Ru, Pt_3_Ru_1/2_Co_1/2_, Pt_3_Ru_1/2_Fe_1/2_ and Pt_3_Ru_1/2_Ni_1/2_.** Electrochemically active surface area (ECSA) for Pt, Pt_3_Ir, Pt_3_Ru, Pt_3_Ru_1/2_Co_1/2_, Pt_3_Ru_1/2_Fe_1/2_ and Pt_3_Ru_1/2_Ni_1/2_ measured from CO stripping voltammograms. CO stripping voltammograms were performed in CO-saturated 0.1 M HClO_4_ aqueous electrolytes with a scanning rate of 50 mV s­^-1^.


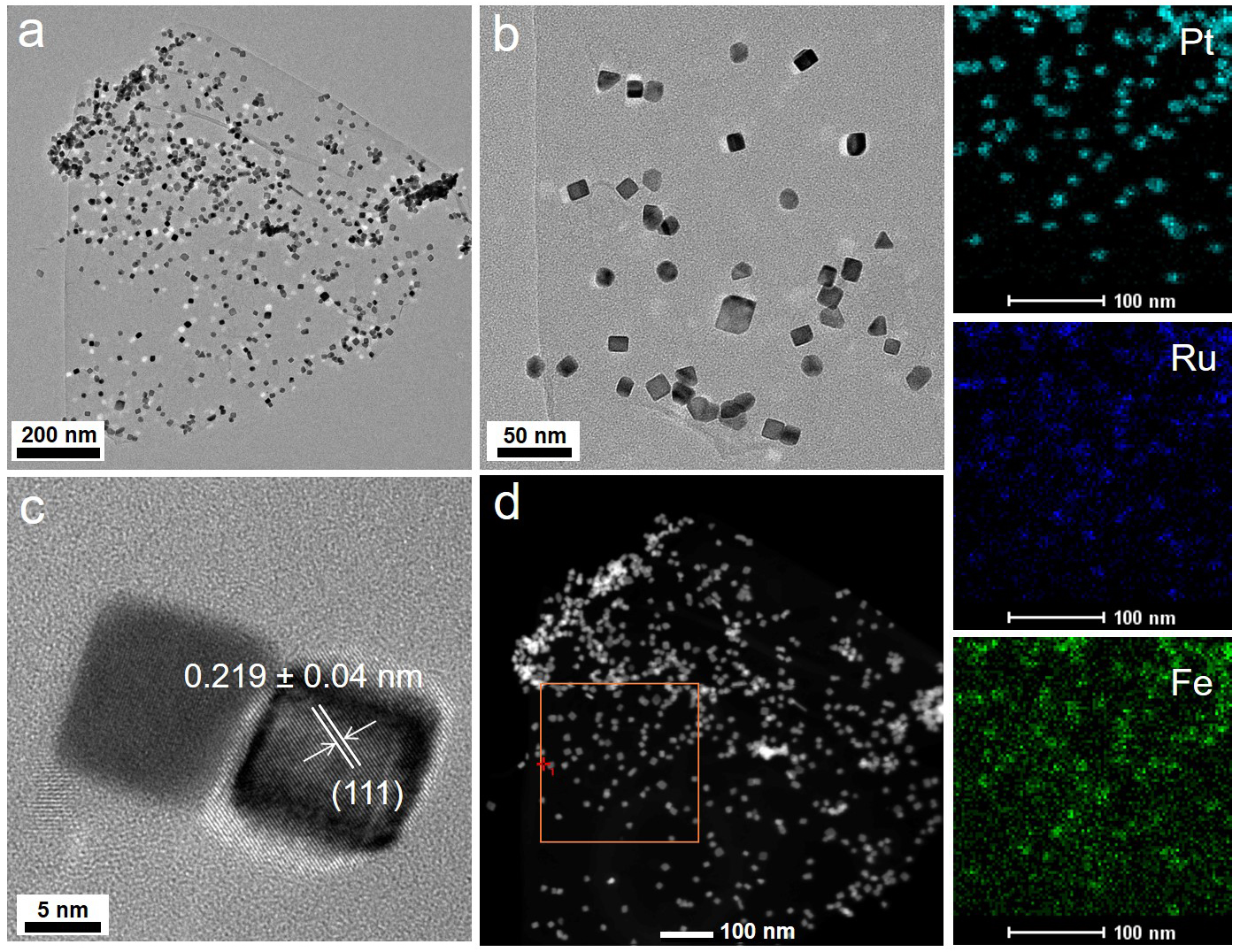


**Supplementary Figure 19| Morphology and structure characterization of the rGO supported Pt_3_Ru_1/2_Fe_1/2_ cubic nanoparticles by TEM.** (**a,b**) TEM, (**c**) HR-TEM, (**d**) HAADF-STEM images and corresponding EDS elemental mapping of Pt, Ru and Fe.


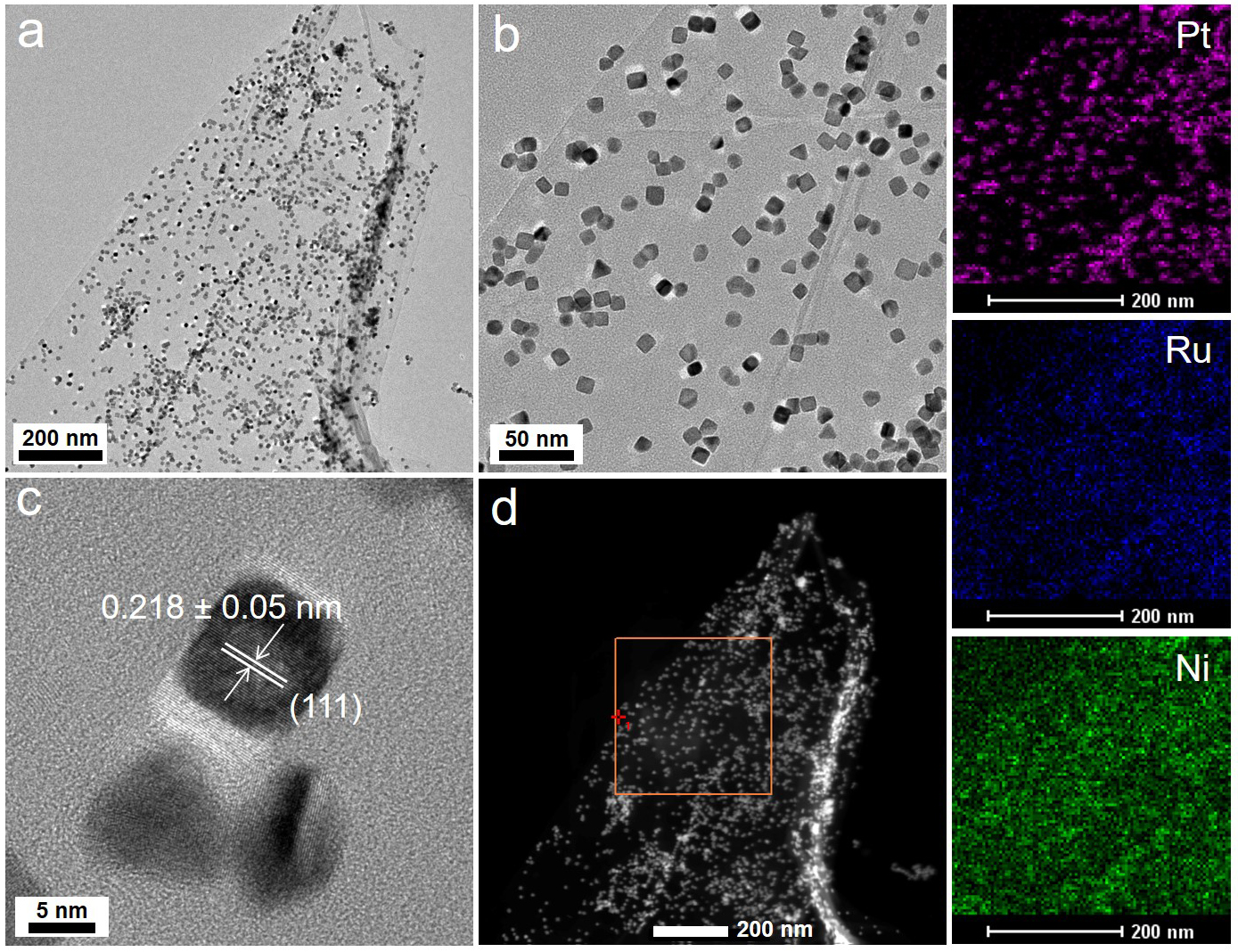


**Supplementary Figure 20| Morphology and structure characterization of the rGO supported Pt_3_Ru_1/2_Ni_1/2_ cubic nanoparticles by TEM.** (**a,b**) TEM, (**c**) HR-TEM, (**d**) HAADF-STEM images and corresponding EDS elemental mapping of Pt, Ru and Ni.

**
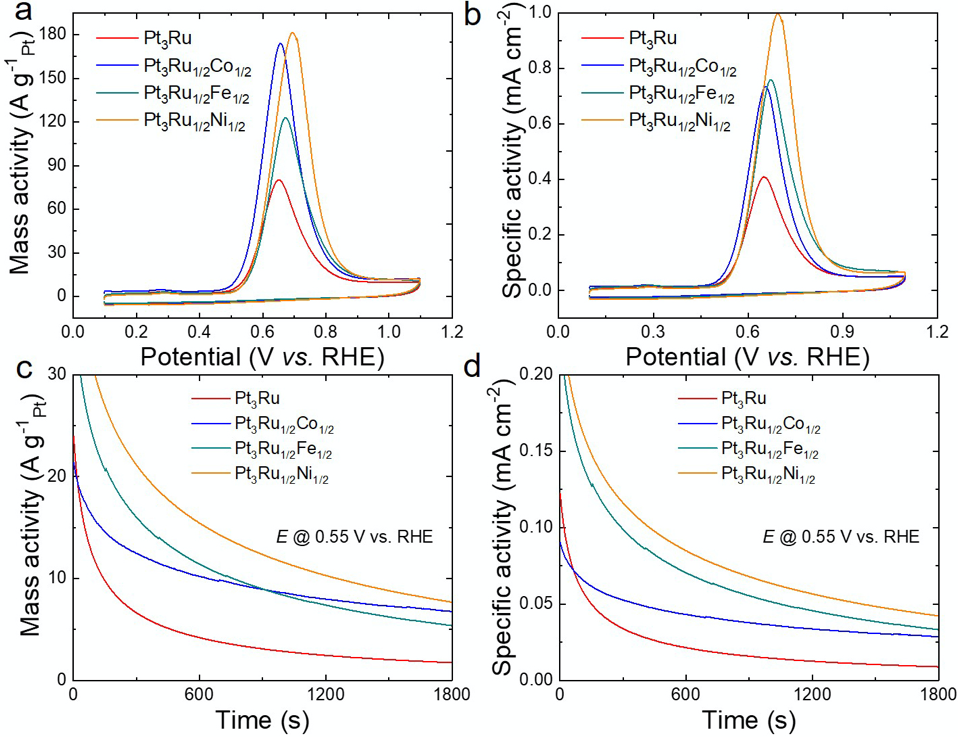
**

**Supplementary Figure 21**| **NH_3_ electrooxidation performance.** (**a, b)** Electrocatalytic performance testing of the Pt_3_Ru, Pt_3_Ru_1/2_Co_1/2_, Pt_3_Ru_1/2_Fe_1/2_ and Pt_3_Ru_1/2_Ni_1/2_ nanocubes via cyclic voltammetry (CV) with a rotating speed of 900 rpm in Ar-saturated 1.0 M KOH + 0.1 M NH_3_ under ambient conditions. (**c, d**) The NH_3_ electrooxidation short-term stability of the Pt_3_Ru, Pt_3_Ru_1/2_Co_1/2_, Pt_3_Ru_1/2_Fe_1/2_ and Pt_3_Ru_1/2_Ni_1/2_ nanocubes at 0.55 V vs. RHE using the same testing conditions. These results suggest further alloying the third metals (i.e., Co, Fe or Ni) could indeed generate an enhanced AOR performance in terms of both mass and specific activities determined by CV and CA measurements.


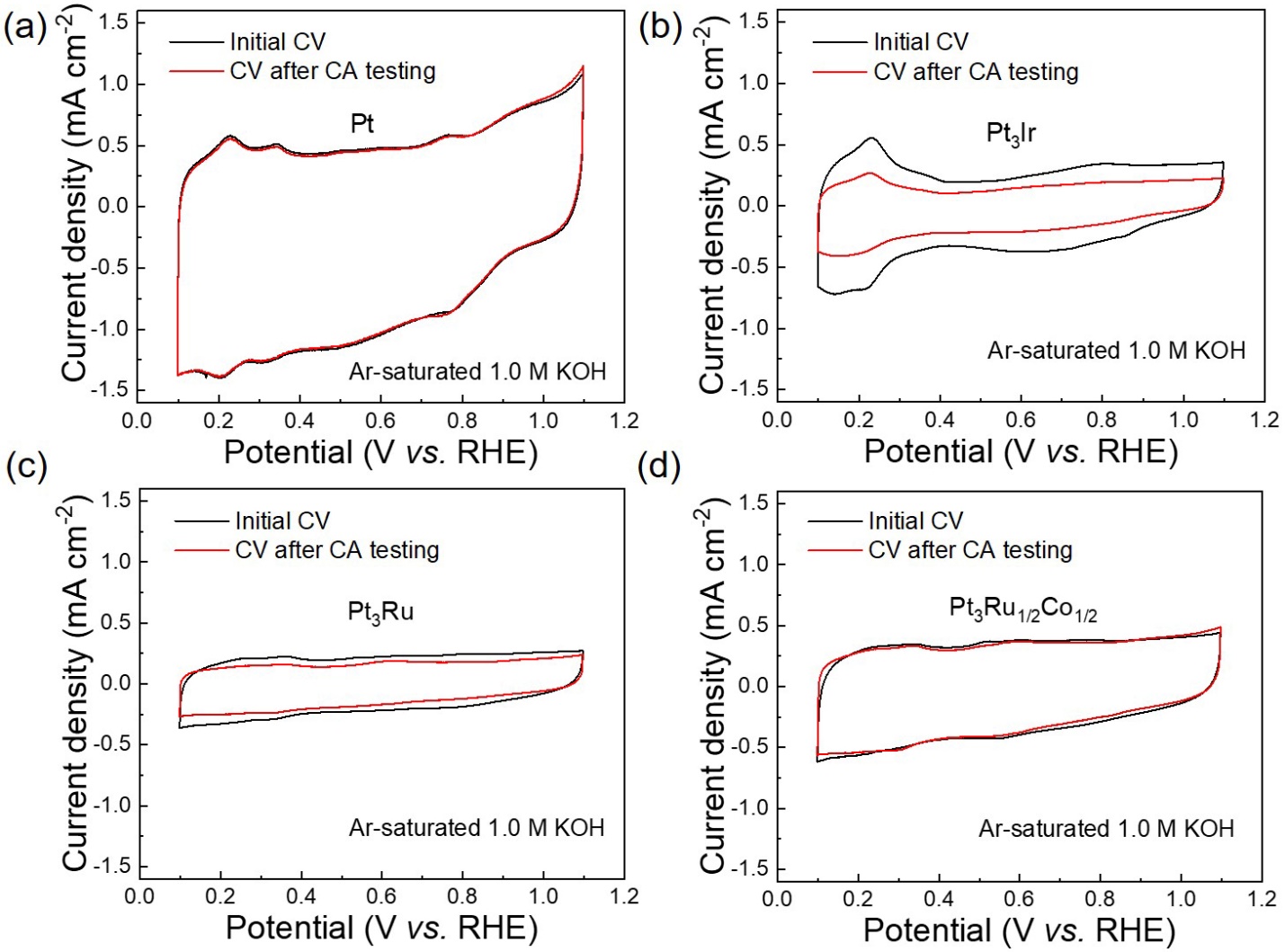


**Supplementary Figure 22| Electrochemical characterization of the Pt, Pt_3_Ir, Pt_3_Ru and Pt_3_Ru_1/2_Co_1/2_ catalysts (a-d).** CV comparisons before and after 3600 s of CA testing (at 0.55 V vs. RHE) in Ar-saturated 1.0 KOH aqueous solution. Note that the catalyst electrodes were electrochemically reduced at a negative potential of –0.135 V *vs.* RHE for five minutes in a fresh 1.0 M KOH aqueous electrolyte after the 3,600 s of CA testing to clean the catalyst surface. These CV comparison results indicate that catalyst deactivation occurs on each studied sample, especially for Pt_3_Ir and Pt_3_Ru, the surface of which cannot be recovered/partially recovered by the electrochemical reduction method. In contrast, the surface of Pt and Pt_3_Ru_1/2_Co_1/2_ can be partially recovered with only smaller changes on their surface.


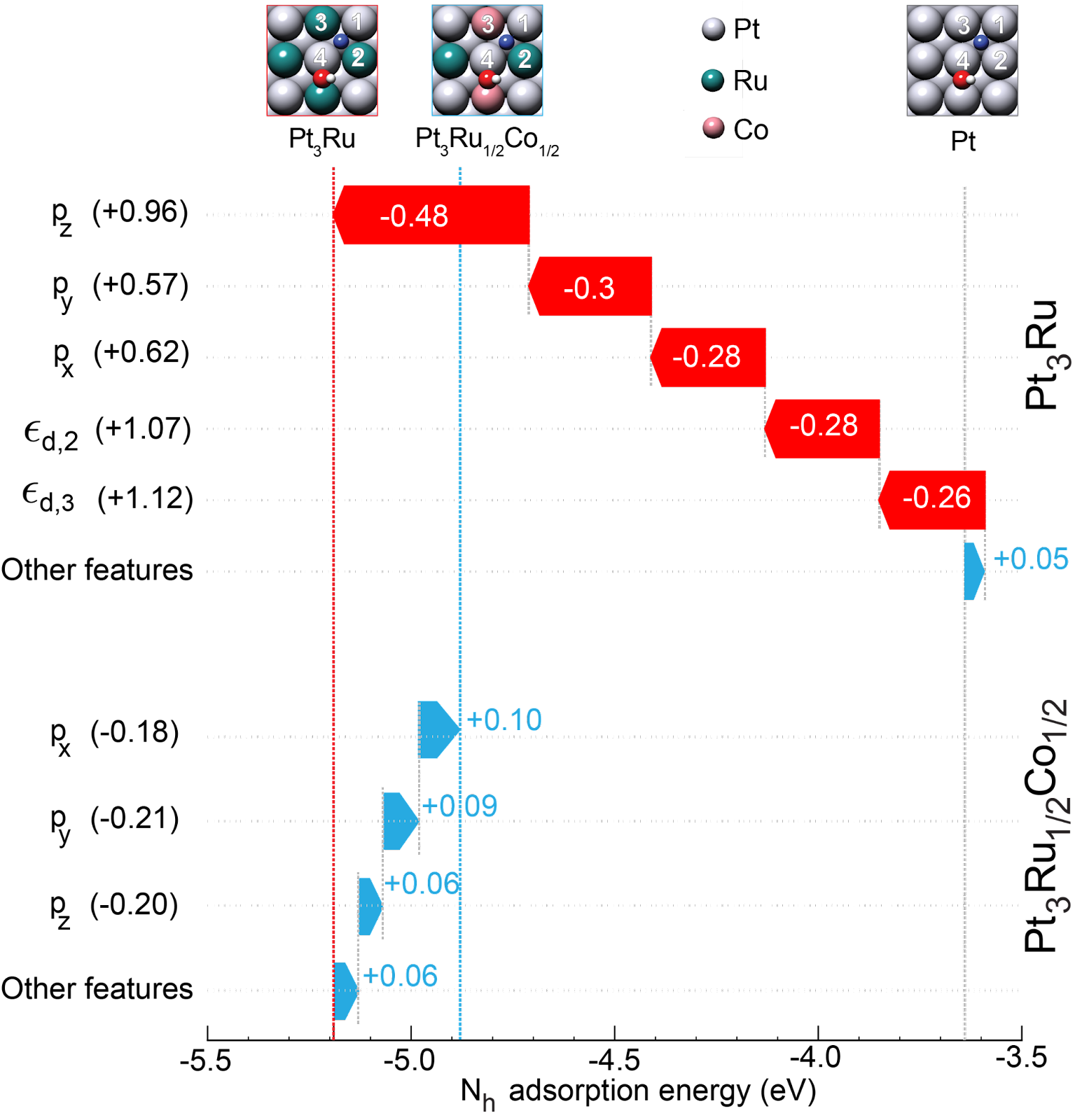


**Supplementary Figure 23| Contribution of individual features from LIME analysis on Pt(100), Pt_3_Ru(100) and Pt_3_Ru_1/2_Co_1/2_(100).** Waterfall plot shows the contribution of each feature from LIME analysis of the TinNet predicted *N hollow binding energy on Pt, Pt_3_Ru and PtRu_1/2_Co_1/2_. The individual feature contributions are taken by calculating the feature effect relative to Pt. A similar conclusion as SHAP is found when using LIME analysis.


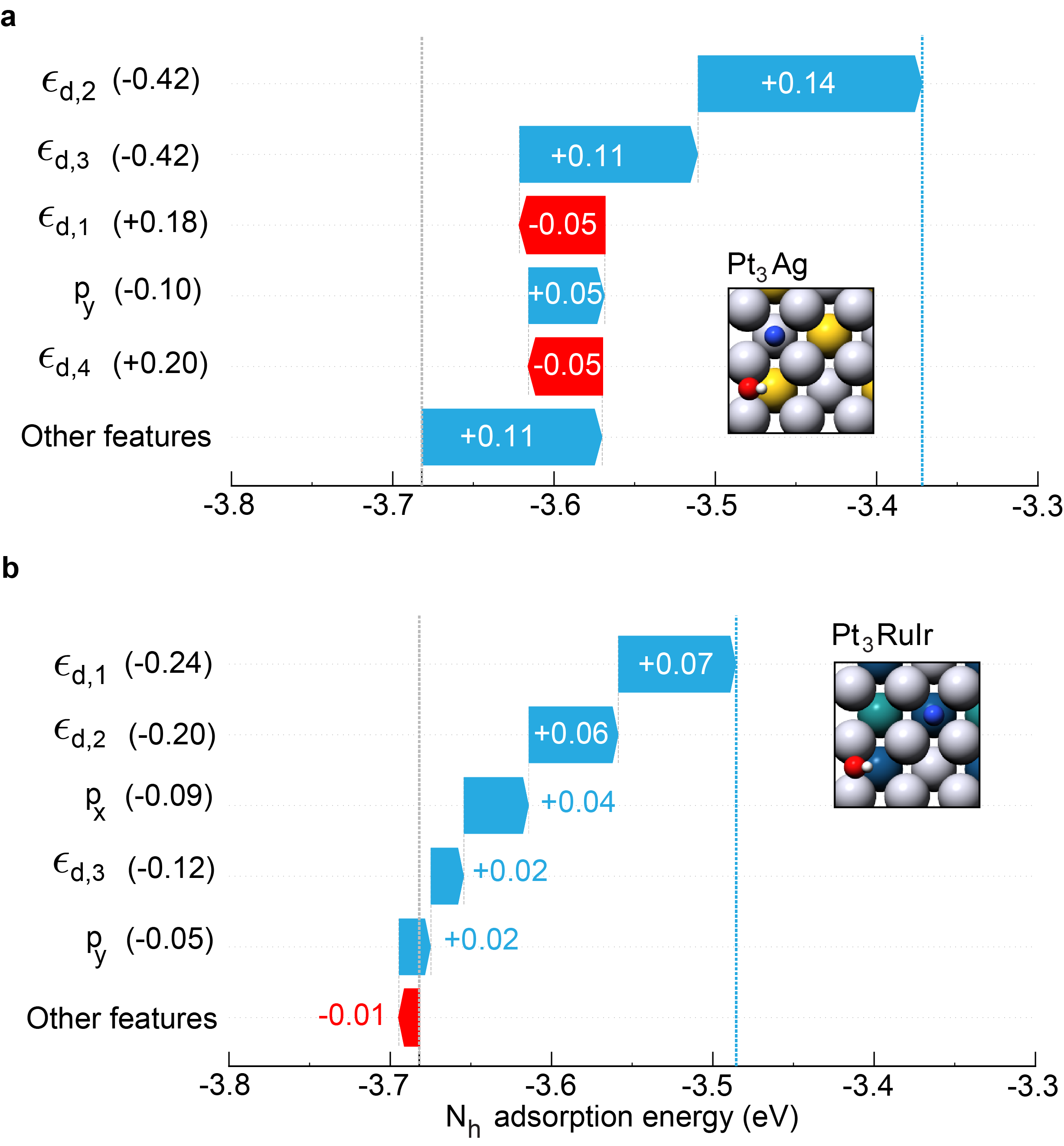


**Supplementary Figure 24| SHAP analysis of *N hollow binding energy on Pt­_3_Ag and Pt_3_RuIr.** SHAP analysis of the TinNet predicted *N hollow binding energy on Pt terminated (**a**) Pt_3_Ag and (**b**) Pt_3_RuIr illustrates systems within the design space where the traditional d-band centre ($\epsilon_{d}$) dominates the reactivity trend.

**References**

1. Wang, S., Pillai, H. S. & Xin, H. Bayesian learning of chemisorption for bridging the complexity of electronic descriptors. *Nat. Commun.* **11**, 6132 (2020).

2. Xie, T. & Grossman, J. C. Crystal Graph Convolutional Neural Networks for an Accurate and Interpretable Prediction of Material Properties. *Phys. Rev. Lett.* **120**, 145301 (2018).

3. Varma, S. & Simon, R. Bias in error estimation when using cross-validation for model selection. *BMC Bioinformatics* **7**, 91 (2006).
